# Identification of myoferlin as a mitochondria-associated membranes component required for calcium signaling in PDAC cell lines

**DOI:** 10.1186/s12964-024-01514-z

**Published:** 2024-02-17

**Authors:** Sandy Anania, Martin Farnir, Raphaël Peiffer, Yasmine Boumahd, Marc Thiry, Ferman Agirman, Naima Maloujahmoum, Akeila Bellahcène, Olivier Peulen

**Affiliations:** 1 Metastasis Research Laboratory, GIGA-Cancer, Pathology Institute B23, Université de Liège, Liège, B-4000 Belgium; 2 STAR Institute, Université de Liège, Allée du 6 Août 19, Liège, B-4000 Belgium; 3 Cellular and Tissular Biology, GIGA-Neurosciences, Cell Biology L3, Université de Liège, Liège, B-4000 Belgium; 4 Center for Interdisciplinary Research on Medicines (CIRM), Mitochondria Adaptation in Cancer Group, Pathology Institute B23, Université de Liège, Liège, B-4000 Belgium

**Keywords:** Pancreatic cancer, Mitochondria-associated membranes, ER-mitochondria contact sites, Myoferlin, Calcium signaling, IP3R3, Mitochondria

## Abstract

**Background:**

Pancreatic ductal adenocarcinoma is an aggressive cancer type with one of the lowest survival rates due to late diagnosis and the absence of effective treatments. A better understanding of PDAC biology will help researchers to discover the Achilles’ heel of cancer cells. In that regard, our research team investigated the function of an emerging oncoprotein known as myoferlin. Myoferlin is overexpressed in PDAC and its silencing/targeting has been shown to affect cancer cell proliferation, migration, mitochondrial dynamics and metabolism. Nevertheless, our comprehension of myoferlin functions in cells remains limited. In this study, we aimed to understand the molecular mechanism linking myoferlin silencing to mitochondrial dynamics.

**Methods:**

Experiments were performed on two pancreas cancer cell lines, Panc-1 and MiaPaCa-2. Myoferlin localization on mitochondria was evaluated by immunofluorescence, proximity ligation assay, and cell fractionation. The presence of myoferlin in mitochondria-associated membranes was assessed by cell fractionation and its function in mitochondrial calcium transfer was evaluated using calcium flow experiments, proximity ligation assays, co-immunoprecipitation, and timelapse fluorescence microscopy in living cells.

**Results:**

Myoferlin localization on mitochondria was investigated. Our results suggest that myoferlin is unlikely to be located on mitochondria. Instead, we identified myoferlin as a new component of mitochondria-associated membranes. Its silencing significantly reduces the mitochondrial calcium level upon stimulation, probably through myoferlin interaction with the inositol 1,4,5-triphosphate receptors 3.

**Conclusions:**

For the first time, myoferlin was specifically demonstrated to be located in mitochondria-associated membranes where it participates to calcium flow. We hypothesized that this function explains our previous results on mitochondrial dynamics. This study improves our comprehension of myoferlin localization and function in cancer biology.

**Supplementary Information:**

The online version contains supplementary material available at 10.1186/s12964-024-01514-z.

## Background

With a 5-year survival rate below 9%, pancreatic ductal adenocarcinoma (PDAC) is an aggressive cancer type [1]. Surgery, the only curative treatment for PDAC, is possible in less than 20% of cases due to late diagnosis [2, 3]. It is estimated that ~ 76% of patients benefiting from surgery will experience a relapse within two years, while for patients diagnosed at late stages (> 80% of cases), only palliative chemotherapy is proposed [3, 4]. Since there is no effective treatment for this cancer type [2, 4], having a better understanding of PDAC biology will help researchers to find the Achilles’ heel of PDAC cancer cells. In that regard, one proposed strategy is to target cancer cell metabolism [5]. In fact, cancer cells adapt their energy metabolism to support their growth and division, as well as challenging environmental conditions such as a lack of nutrients and hypoxia [6, 7]. Consequently, disrupting cancer cell metabolism, to create a metabolic imbalance, could slow down cell proliferation and sensitize them to other therapies.

Over the last few years, our research team has investigated the function of a protein known as myoferlin. This protein is overexpressed in PDAC and its silencing/targeting has been shown to affect cancer cell metabolism, proliferation and migration in vitro [8–11]; small compounds targeting myoferlin reduced the number of metastases and tumor size in mouse models [12–14]. Furthermore, it increased overall survival, with no apparent toxic side effects in mice [12–14]. Therefore, myoferlin targeting seems to be a promising therapeutic approach. Nevertheless, our comprehension of myoferlin functions in cells remains limited and the underlying mechanisms explaining the effect of myoferlin targeting are still unknown. For instance, it has been reported that myoferlin silencing leads to a fragmented mitochondrial network and reduced mitochondrial respiration [8]. However, no conclusive mechanisms have been reported yet.

Since mitochondria are involved in PDAC relapse and tumor growth, we aimed to understand the mechanism linking myoferlin silencing to mitochondrial dynamics [15, 16]. Based on our recent report showing an interaction between myoferlin and mitofusin [17], we first hypothesized that myoferlin was directly involved in mitochondrial dynamics by being present on the outer membrane of this organelle. The present report suggests that myoferlin is unlikely located on mitochondria but in the membranes associated with this organelle such as lysosomes or endoplasmic reticulum (ER). Initially, we investigated the presence of myoferlin in the contacts existing between ER and mitochondria. In the literature, those contacts are named mitochondria-associated membranes (MAMs) or mitochondria-ER contact sites (MERCS) and are known to influence cell metabolism and fate. MAMs have been described in many cellular processes including mitochondrial dynamics, apoptosis, lipid synthesis, calcium (Ca^2+^) transfer, autophagy and inflammation [18, 19]. In addition, this metabolic platform has been associated with pathologies such as cancer, diabetes, Alzheimer, and Parkinson disease [20, 21].

Our results show that myoferlin is indeed present in MAMs and its silencing significantly reduces the mitochondrial calcium level upon stimulation, probably through an interaction with inositol 1,4,5-triphosphate receptor 3 (IP3R3). This discovery improves our comprehension of myoferlin localization and function in cancer biology.

## Methods

### Cells and chemicals

The investigations performed in this report were based on PDAC cell lines (Panc-1 and MiaPaCa-2). Panc-1 (CRL-1469) and MiaPaCa-2 (CRL-1420) were generous gifts from Prof. Muller and Burtea (NMR Laboratory, University of Mons, Belgium) and Prof. De Wever (Laboratory of Experimental Cancer Research, University of Gent, Belgium), respectively. We selected these cell lines as representative members of the lipogenic (Panc-1) and the glycolytic (MiaPaCa-2) subgroups of PDAC cell lines [22]. Antibodies against the 75 KDa glucose-regulated protein (GRP75, clone D13H4, #3593), mitochondrial import receptor subunit TOM20 homolog (TOM20, clone D8T4N, #42,406), calreticulin (clone D3E6, #12,238), glucose transporter 1 (GLUT1, #12,939), binding immunoglobulin protein (GRP78/BIP, #3177), inositol requiring enzyme 1 (IRE1, #3294), X-box-binding protein-1 (XBP1s, #12,782), PKR-like ER kinase (PERK, #5683), activating transcription factor 4 (ATF4, #11,815) and transcription factor C/EBP-homologous protein (CHOP, #2895) were from Cell Signaling (Danvers, MA). Vinculin (sc-25,336), myoferlin (clone D-11, sc-376,879), sigma-1 receptor (S1R, sc-137,075), specificity protein 1 (SP1, sc-17,824), and heat shock cognate 71 kDa protein (HSC70, sc-7298) antibodies were purchased from Santa-Cruz Biotechnology (Dallas, TX). The 78 kDa glucose-regulated protein (GRP78/BIP, MAB4846) and mitochondria (Clone 113-1, MAB1273) antibodies were obtained from R&D systems (Minneapolis, MN) and Millipore (Burlington, MA), respectively. A second antibody against myoferlin (identified here under as HPA - HPA014245) was from Sigma (Bornem, Belgium). The voltage-dependent anion channel 1 (VDAC1, clone 20B12AF2, ab14743), the total OXPHOS cocktail (ab110413), glyceraldehyde-3-phosphate dehydrogenase (GAPDH, ab8245) and mitofusin 1/2 antibodies (MFN1/2, clone 3C9, ab57602) were from Abcam (Cambridge, UK). Antibody against IP3R3 (PA5-88758) was obtained from Invitrogen (Waltham, USA). The antibodies used for the unfolded protein response (UPR) were a generous gift from Dr. Arnaud Blomme (GIGA stem cells, ULiège). All other reagents were purchased from Sigma (Bornem, Belgium), unless mentioned otherwise.

### Cell culture

Panc-1 cells were cultured in Dulbecco’s modified Eagle’s medium (DMEM, Lonza, Basel, Switzerland) supplemented with 10% fetal bovine serum (FBS), 1X non-essential amino acids and 2 mM L-glutamine. Miapaca-2 were maintained in DMEM supplemented with 10% FBS, 1 mM sodium pyruvate, and 4 mM L-glutamine. All cells were cultured in a humidified 5% CO_2_ incubator, at 37 °C and were used between passage 1 and 10. Mycoplasma contamination was checked monthly by analysing the activity of mycoplasma enzymes (acetate kinase and carbamate kinase) in cell culture media. In brief, enzyme substrates (3 mM acetyl phosphate, 3 mM carbamoyl phosphate), 5 µM ADP, 2 mM AMP, 3 mM magnesium acetate, 3 mM inorganic pyrophosphate, and 0,25% Triton X100 were added to 50 µl culture media. The resulting ATP production was assessed by firefly luciferase activity.

### Small interfering RNA transfection

Cells were transfected with 20 nM small interfering RNA (siRNA) using Ca^2^^+^ phosphate. The medium was replaced 16 h after transfection, media replacement was considered as time 0. All experiments were performed 48 h after transfection. Myof#1 (5’-CCCUGUCUGGAAUGAGAUUUU-3’) and Myof#2 (5’-CUGAAGAGCUGUG-CAUUATT-3’) siRNA were used to deplete myoferlin, while the firefly luciferase siRNA (5’-CUUACGCUGAGUACUUCGAUU-3’ - identified here under as irrelevant siRNA) was used as the transfection control. All siRNAs were manufactured by Eurogentec (Seraing, Belgium).

### Plasmid preparation and transfection

CMV-mito-R-GECO1 was constructed by Robert Campbell (Addgene plasmid # 46,021) [23]. The plasmid was amplified in DH10B bacteria. Bacteria were cultured in LB medium, supplemented with ampicillin (100 µg/mL), overnight at 37 °C in an orbital incubator shaker (200 rpm). Purification was performed using the NucleoBond Xtra Maxi kit (#740424.50) and the NucleoSnap Finisher kit (#740434.50) from Macherey Nagel (Düren, Germany) with the help of the GIGA viral vectors platform. Panc-1 or MiaPaCa-2 cells were transiently transfected with 1 µg of plasmid using 2.5 µL Lipofectamine 2000 (Invitrogen, Carlsbad, CA) as reported by the manufacturer. The medium was replaced 4 h after transfection. 48 h after transfection, both Panc-1 and MiaPaCa-2 cells were selected with 600 µg/mL of G-418 for 7 days. Antibiotic pressure using G-418 solution was maintained at a concentration of 200 µg/mL for cell culture. To address CMV-mito-R-GECO1 plasmid localization on mitochondria, we used a MitoTracker Green dye (M7514, Thermo Fisher Scientific, Waltham, USA), at a final concentration of 200 nM, which was a generous gift from Dr. Laurent Nguyen (GIGA Stem Cells, ULiège).

### Western blotting

Protein samples were solubilized in 1% sodium dodecyl sulfate (SDS) supplemented with phosphatase and protease inhibitors. A bicinchoninic acid protein assay kit (Thermo Scientific, Waltham, MA) was used for protein quantification. Proteins were denatured in Laemmli’s buffer for 5 min at 99 °C. Samples were loaded on SDS polyacrylamide gel for migration and were then electrotransferred on a PVDF membrane during 90 min at room temperature (RT) or overnight at 4 °C. Membranes were blocked for 1 h according to the antibody manufacturers’ instructions. Then, membranes were incubated overnight at 4 °C with primary antibodies (dilution 1:1000) and probed with corresponding secondary antibodies conjugated to horseradish peroxidase (dilution 1:3000) for 1 h at RT. Revelation was performed using chemiluminescent reagents (ECL western blotting substrate, Thermo Scientific, Waltham, MA or clarity western ECL substrate, Bio-Rad, California, USA). Quantifications were performed by densitometric analysis using ImageJ software [24] and HSC70 was used as loading control.

### Immunofluorescence

Cells (6 × 10^4^) were seeded on sterilized glass coverslips. After 24 h, cells were washed once with PBS and fixed with paraformaldehyde 4% (pH7.4) for 20 min. Then, cells were washed twice with PBS and were blocked and permeabilized for 30 min with a solution containing 5% BSA and 0.5% saponin in PBS. After blocking, coverslips were incubated for 2 h with primary antibodies (dilution 1:100 in BSA-PBS) at RT in a humidified chamber. Antibodies were diluted in 1% BSA − 0,1% saponin - PBS solution. This step was followed by three washes in 1% BSA-PBS. Coverslips were then incubated with corresponding Alexa Fluor 488 or Alexa Fluor 546 conjugated secondary antibodies (Invitrogen, Molecular Probes, Carlsbad, CA) in a humidified chamber for 45 min (dilution 1:1000 in BSA-PBS). Nuclei counterstaining was performed using a hoechst DNA dye (0.01 g/L, Calbiochem, San Diego, CA). Pictures were acquired using a Nikon A1R confocal microscope or LSM880 Airyscan Elyra Microscope (Zeiss, Oberkochen, Germany).

### Colocalization studies

We used Manders’ method to assess myoferlin localization on mitochondria. Since we observed that the Costes automatic threshold estimation was often too low, leading to the inclusion of non-specific signals, we wrote a script based on the JACoP ImageJ plugin to standardize the results and make them comparable [25]. This was enforced by keeping consistent threshold values throughout the set of images. In addition, individual cells were used as a region of interest (ROI).

### Proximity ligation assay

The Duolink proximity ligation assay (PLA) kit (DUO92008, Sigma, Bornem, Belgium) was used according to the manufacturer’s instructions. Primary antibodies were used at dilution 1:75 (Myoferlin, MFN1/2) or 1:100 (others). Oligonucleotide conjugated secondary antibodies were provided by the kit allowing the detection of a red signal if less than 40 nm separate both proteins of interest. Pictures were acquired using Nikon A1R confocal microscope. In each microscopic field, proximity dots were counted using ImageJ software [24] and divided by the number of nuclei to calculate an average proximity dots number per cell. To analyze PLA, we wrote a script to automatically analyze the data in a standardized way. The script is available at [26].

### Co-immunoprecipitation

Proteins were extracted using a non-denaturing buffer containing Tris-HCl (pH8, 20 mM), NaCl (137 mM), NP40 (1%), EDTA (2 mM) and supplemented with protease inhibitors. Following extraction, proteins were incubated under rotation at 4 °C for 30 min and centrifuged at 14,000 g for 15 min at 4 °C to eliminate cell debris. 5 µg antibodies were incubated overnight with 500 µg of the protein extract (except for the IP performed from MAMs extracts, where 250 µg were incubated). We used isotype IgG as a control (Thermo Scientific, Waltham, MA). Then, protein A/G magnetic beads (Thermo Scientific, Waltham, MA) were added and incubated at 4 °C under rotation for 2 h. After three washes with a low salt buffer containing SDS (0.1%), Triton X-100 (1%), EDTA (2 mM), Tris-HCl pH 8 (20 mM) and NaCl (150 mM) and one wash of high salt buffer composed of SDS (0.1%), Triton X-100 (1%), EDTA (2 mM), Tris-HCl pH 8 (20 mM) and NaCl (450 mM), proteins were eluted at 99 °C from magnetic beads using Laemmli’s buffer and then processed for western blotting.

### Subcellular fractionation using percoll gradient

This experiment was based on the protocol published by Lewis et al. [27]. 8 × 10^6^ cells were seeded on 150 mm dishes and placed in an 5% CO_2_ incubator, at 37 °C. Approximately 40 dishes at 80% confluency per experiment were used. 24 h after seeding, the cells were placed on ice and washed once with ice-cold PBS. Then, they were detached from the dishes by scrapping and centrifuged for 5 min at 500 g. The resulting pellet was suspended in HB buffer (10 mM HEPES, pH7.4, and 0.25 M sucrose) and homogenized with a Teflon glass homogenizer for 30 strokes. After centrifugation (5 min, 600 g), the supernatant was kept aside (supernatant 1) and the pellet was again suspended in HB buffer, homogenized for 15 strokes and centrifuged for 5 min at 600 g. The resulting supernatant was pooled with supernatant 1. The pellet constituted the P1 fraction, representing non-lysed cells, nuclei, and cell debris. Supernatant 1 was then centrifuged for 20 min at 10,300 g. The resulting supernatant (supernatant 2) was ultracentrifuged for 60 min at 100,000 g, giving cytosolic (supernatant) and microsome (pellet) fractions. In parallel, the pellet obtained by centrifugation from supernatant 1, constituting crude mitochondrial fraction (CM), was resuspended in IM buffer (5 mM HEPES, pH7.4, 250 mM mannitol, 0.5 mM EGTA) and placed on percoll (25 mM HEPES, pH7.4, 225 mM mannitol, 1 mM EGTA, 30% Percoll (v/v)). Following this step, centrifugation for 30 min at 95,000 g was performed. MAMs and mitochondria formed a white layer near the top of the tube and were collected using Pasteur pipettes. Then, MAMs were suspended in IM2 buffer (25 mM HEPES, pH7.4, 225 mM mannitol, 1 mM EGTA) and centrifuged for 10 min at 6,300 g. On the one hand, the pellet was harvested as crude MAMs (CMAMs). On the other hand, the resulting supernatant was centrifuged for 1 h at 100,000 g. The white membrane at the bottom of the tube following centrifugation was harvested as purified MAMs fraction (PMAMs). In parallel, mitochondria obtained from centrifugation on percoll were washed three times with the IM buffer. The pellet obtained from washing constituted purified mitochondrial fraction (PM). During the experiments, samples were kept on ice and all centrifugations were performed at 4 °C.

### Ultrastructural analysis

Panc-1 cells were fixed for 90 min at room temperature with glutaraldehyde (2.5%) in a Sörensen phosphate buffer (0.1 M, pH7.4) and postfixed for 30 min with 2% osmium tetroxide. Samples were dehydrated in graded ethanol and embedded in Epon. Thanks to a Reichert Ultracut S ultramicrotome, ultrathin sections were obtained and contrasted with uranyl acetate and lead citrate. Acquisitions were performed using a Jeol (Tokyo, Japan) JEM-1400 transmission electron microscope at 80 kV. Morphometric measurements were performed using ImageJ software [24]. The length of ER interface in contact with mitochondria, the mitochondrial perimeter and the distance between both organelles were measured. For calculations of mitochondria–ER distance, a minimum distance of 30 nm between both organelles was required to be consider as a contact. To evaluate the extent of contact between the ER and mitochondria, we referred to the ER-mitochondria contact coefficient (ERMICC) described by Naon et al. [28]. This coefficient relies on three parameters: the length of ER-mitochondria interface (Lin), the distance between mitochondria and ER (DistER-M), and the mitochondrial perimeter (PerM). The ERMICC is defined as followed:

$$ERMICC=\frac{{L}_{in}}{{Per}_{M}*{Dist}_{ER-M}}$$

### Calcium flow

Cells stably transfected with the CMV-mito-R-GECO1 plasmid (200,000 cells) were seeded on glass-bottom dishes (Ibidi, 81,158, Gräfelfing, Germany) 24 h before to experiment. The next day, the cells were washed once with PBS and the medium was replaced by calcium-free medium (145 mM NaCl, 4 mM KCl, 10 mM HEPES, 10 mM glucose, 2 mM MgCl_2_ and 1 mM EGTA). Then, the dishes were placed on a Nikon A1R microscope stage in a humidified chamber with 5% CO_2_, at 37 °C. 10 min after temperature stabilization, pictures acquisition occurred every 5 s (a cycle represents the time between two acquisitions). After 50 s, a calcium-free buffer containing histamine was injected (100 µM final concentration). Pictures acquisition continued for 50 additional cycles until the fluorescence returned to its starting value.

For the analyses, we assessed the fluorescence intensity value over time (Fn) for each cell and normalized it to the first frame (F0) of the timelapse (Fn/F0), which allows comparisons between conditions. These analyses were performed using ImageJ [24]. A script, which is available on [29], was written to automate and standardize the analyses.

### Statistical analysis

For parametric analyses, according to the number of experimental conditions to compare, unpaired t-test or one-way analysis of variance were performed. For multiple comparisons, Dunnett or Tukey tests were applied. When the data were not following a normal distribution, non-parametric tests were conducted. The test of Mann-Whitney was used to compare two groups, while the Kruskal-Wallis’s test was used to compare more than two groups. A *p*-value < 0.05 was considered as statistically significant.

## Results

### Myoferlin is not located on mitochondria in PDAC cell lines

**Fig. 1:**
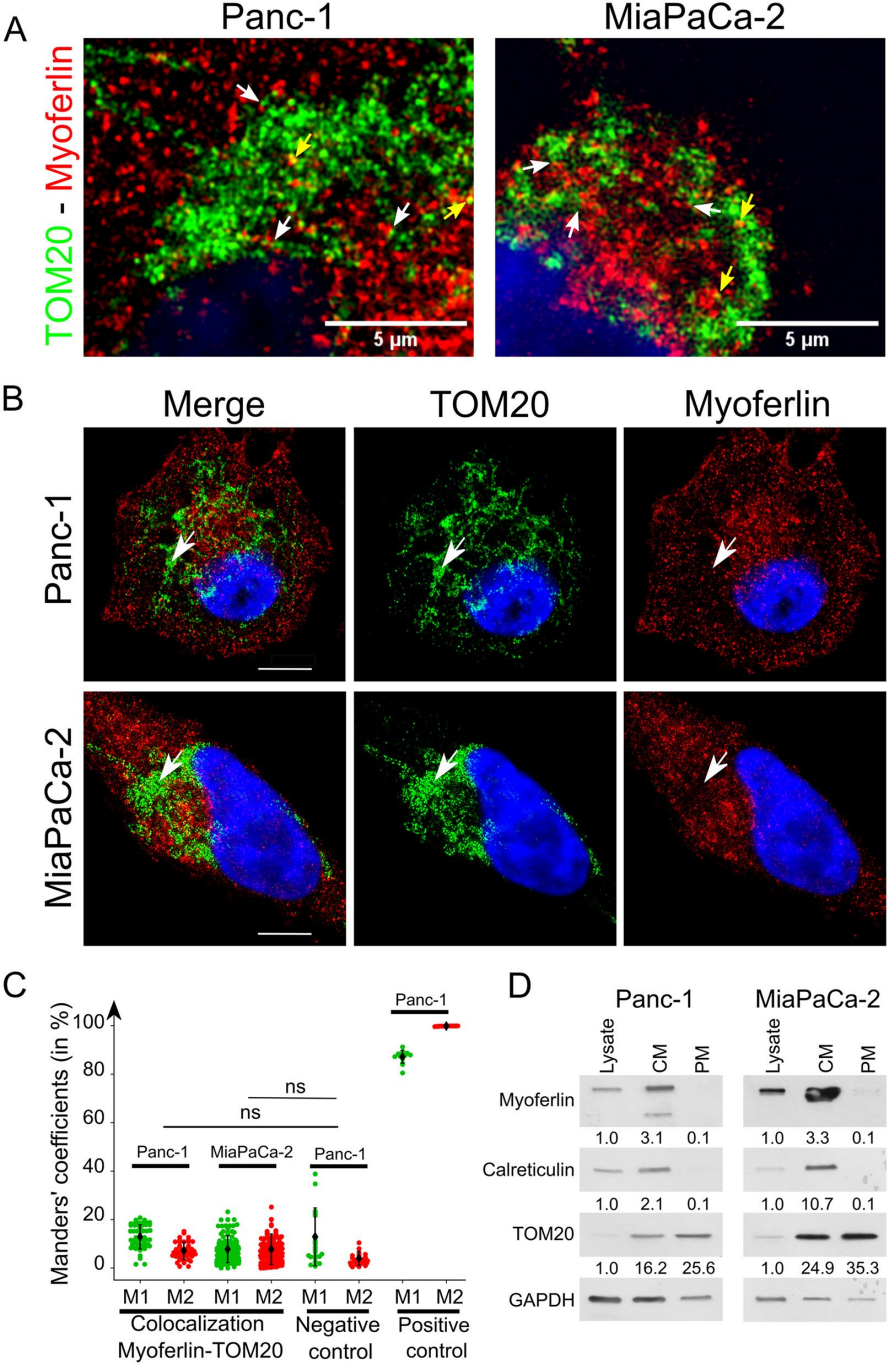
Myoferlin and TOM20 poorly colocalize.** A-C** Indirect immunofluorescence performed on Panc-1 and MiaPaCa-2 cell lines, using TOM20 rabbit monoclonal and myoferlin (D-11) mouse monoclonal antibodies (**A**) High magnification showing myoferlin proximity (white arrows) with TOM20 in Panc-1 and MiaPaCa-2 cell lines. Colocalized pixels are pointed with yellow arrows. Scale bars represent 5 μm. **B** Low magnification showing myoferlin and TOM20 localization within Panc-1 and MiaPaCa-2 cells. White arrows pointed at region with high TOM20 staining while myoferlin staining was low. Scale bar represents 8.89 μm in Panc-1 and 5 μm in MiaPaCa-2 cell lines. The confocal pictures were acquired with a high resolution LSM 880 microscope. **C** Quantification of colocalization between TOM20 and myoferlin using Manders’ method. M1 represents the proportion of TOM20 colocalizing with myoferlin, while M2 represents the proportion of myoferlin colocalizing with TOM20. For the positive control, two secondary antibodies carrying distinct fluorochromes (Alexa Fluor 488 and 546), recognized the same myoferlin rabbit polyclonal primary antibody (HPA). The experiment was performed as three biological replicates. Each dot represents one individual cell. The non-parametric test of Kruskal-Wallis was performed for statistical analyses. Results are presented as mean ± SD, ns: non-significant. **D** Western blot showing myoferlin abundance in mitochondrial fractions. Calreticulin was used as an ER marker, while TOM20 was used as a mitochondrial marker. GAPDH was used as a loading control. These western blots are representative of three biological replicates

To clarify whether myoferlin was located or not on mitochondria, we first performed immunofluorescence co-staining in two PDAC cell lines, Panc-1 and MiaPaCa-2. We used an antibody targeting TOM20 to highlight mitochondria. As shown by the white arrows in Fig. 1A, myoferlin was in proximity with TOM20 in both Panc-1 and MiaPaCa-2 cell lines. However, visual colocalizations (represented by yellow pixels and pointed by yellow arrows) between TOM20 and myoferlin were only occasional. Pictures at low magnification and controls are shown in Figure S1 (Additional file 1). Moreover, as highlighted by the white arrows in Fig. 1B, the myoferlin-labeled pixels were less frequent in the regions labeled for mitochondria (TOM20). Even if the labeling of myoferlin and TOM20 intertwined in some areas, myoferlin and TOM20 did not seem to be located in the same subcellular regions.

Because the visual assessment of colocalization is often biased by signal intensity, we conducted colocalization studies based on Manders’ coefficients [24, 30, 31]. Our results showed that the percentage of TOM20 colocalizing with myoferlin (M1) in the Panc-1 cell line was 12.82 ± 5.02%, whereas in MiaPaCa-2, this percentage was 7.71 ± 5.61% (Fig. 1C). Similarly, the percentage of myoferlin colocalizing with TOM20 (M2) was 7.16 ± 3.56% in Panc-1 and 7.66 ± 6.32% in MiaPaCa-2 (Fig. 1C). The descriptive statistics are presented in Table S1 (Additional file 1). To allow us to quantify the expected range of variations in colocalization coefficients, we included a negative control using SP1 (nucleus) and GLUT-1 (plasma membrane) and a positive control, where two secondary antibodies, carrying distinct fluorochromes, recognized the same myoferlin primary antibody (HPA) (Figure S2, Additional file 1). The Manders’ coefficients from myoferlin-TOM20 pictures were not significantly different than those from SP1-GLUT-1 pictures (Fig. 1C). Moreover, both myoferlin-TOM20 and SP1-GLUT1 co-labeling displayed a low percentage of colocalization compared to the positive control. We thus hypothesized that myoferlin is close to mitochondria rather than directly located on this organelle. To further validate this hypothesis with an additional mitochondrial protein, we decided to perform a PLA between myoferlin and a 60–65 kDa protein restricted to the outer mitochondrial membrane (OMM) (Figure S3, Additional file 1). We were unable to show any PLA colocalization dots in the MiaPaCa-2 cell line, while a few dots were detected in the Panc-1 cell line.

To clarify the presence of myoferlin on mitochondria, we isolated this organelle from Panc-1 and MiaPaCa-2 cell lines using differential and percoll gradient centrifugations (Fig. 1D). We observed an enrichment of TOM20 in the pure mitochondrial (PM) fraction (by PM, we mean fraction of mitochondria not associated with organelles such as ER) compared to the whole cell lysate, confirming the presence of mitochondrial proteins in PM. The enrichment ratio was > 25 and > 35 in Panc-1 and MiaPaCa-2 cell lines, respectively. Calreticulin, a marker of the ER, was almost undetectable in the PM fraction of both Panc-1 and MiaPaCa-2. We noticed that myoferlin was undetectable in the Panc-1 PM fraction and only barely detectable in MiaPaCa-2 PM fraction while enriched in the CM fraction (fraction of mitochondria still associated with organelles such as ER). The enrichment ratio was > 3 in both Panc-1 and MiaPaCa-2 cell lines. Altogether, our results suggest that myoferlin is not located on mitochondria but is rather in proximity with this organelle.

### Myoferlin is found in mitochondria-associated membranes in PDAC cell lines

**Fig. 2:**
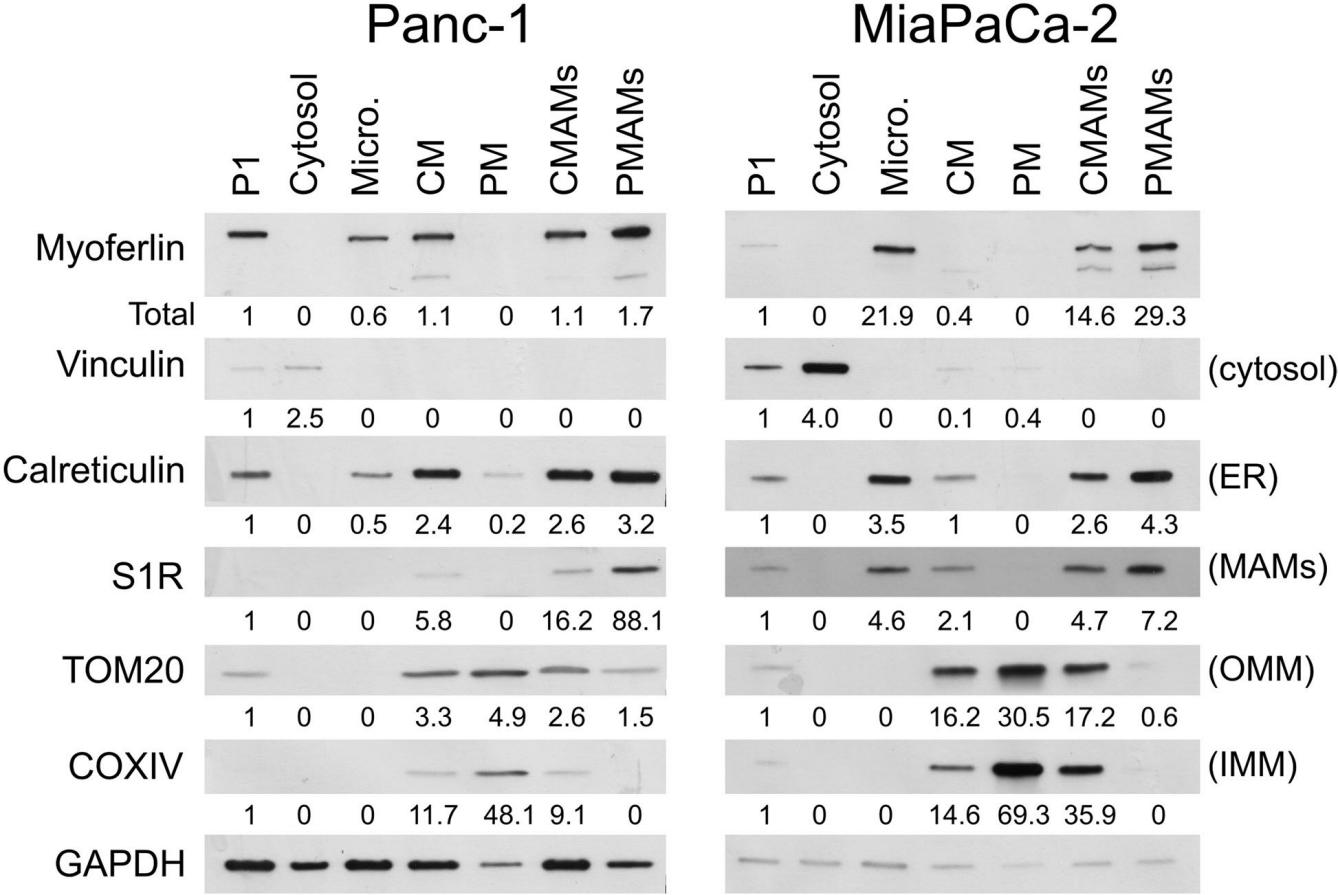
Myoferlin is detected in MAM fractions. Panc-1 and MiaPaCa-2 cells were fractionated in seven fractions: whole cell lysate (P1), cytosol, microsomes (Micro), crude mitochondria (CM), pure mitochondria (PM), crude MAMs (CMAMs), pure MAMs (PMAMs). Vinculin was used as a cytosolic marker, calreticulin as an ER marker, S1R as a MAM marker, TOM20 as an OMM maker and COXIV as an IMM marker. GAPDH was used as a loading control

Based on the proximity between myoferlin and OMM proteins, as well as its absence in mitochondrial fraction, we thought myoferlin may be located in mitochondria-associated membranes (MAMs). Consequently, we performed a cell fractionation allowing pure MAMs (PMAMs) isolation from both Panc-1 and MiaPaCa-2 cell lines [27]. To assess the quality of the fractions, we estimated the abundance of specific markers [27, 32]: vinculin as a cytosolic marker, calreticulin as ER marker, TOM20 as OMM marker, Cytochrome c oxidase subunit 4 (COXIV) as inner mitochondrial membrane (IMM) marker, and S1R as a marker of MAMs. S1R was enriched in the PMAM fractions, with an enrichment ratio > 88 and > 7 in Panc-1 and MiaPaCa-2 cell lines, respectively. These markers validated the quality of the MAMs extraction. We then evaluated myoferlin’s presence in the fractions (Fig. 2). In agreement with previous reports, myoferlin was found in the microsomal fraction [33]. Interestingly, myoferlin was detected in the CMAM and PMAM fractions of both cell lines (Fig. 2). The myoferlin enrichment ratio was particularly elevated in MiaPaCa-2 (ratio > 29). Our results demonstrate the presence of myoferlin in MAMs.

### Myoferlin silencing impacts mitochondrial Ca^2^^+^ flux upon histamine stimulation

Because myoferlin displays a rare structure with multiple calcium-binding C2 domains and impacts mitochondrial metabolism and dynamics upon silencing, we thought this protein could be involved in Ca^2^^+^ signaling at MAMs [34–36]. In order to monitor mitochondrial Ca^2^^+^ levels in an intensiometric manner, we took advantage of the CMV-Mito-R-GECO-1 plasmid encoding a calmodulin-RFP fusion protein harboring a mitochondrial import signal [23]. First, to validate the mitochondrial localization of the fusion protein, we used a MitoTracker probe to highlight mitochondria. We demonstrated, a perfect colocalization between the red fusion protein and MitoTracker Green probe in both Panc-1 and MiaPaCa-2 (Figure S4, Additional file 1).

**Fig. 3:**
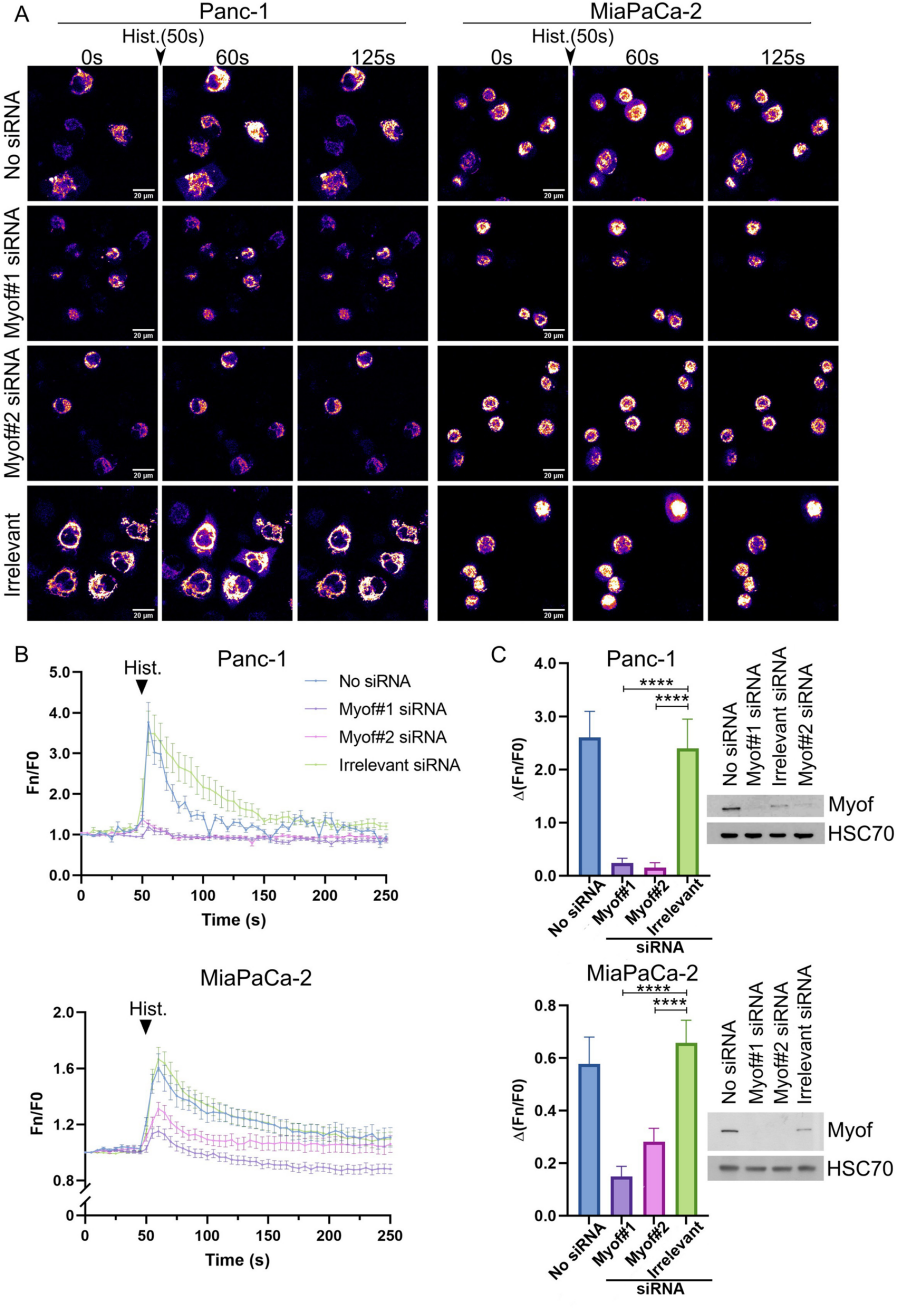
Myoferlin silencing impacts mitochondrial Ca^2^^+^ level upon histamine stimulation. **A** Panc-1 and MiaPaCa-2 cell lines transfected with CMV-Mito-R-GECO-1 plasmid upon histamine stimulation in a Ca^2+^-free medium at different time points (0, 60, 125 s). Histamine injection was done at time point 50 s. Images were acquired with a Nikon A1R microscope. **B** Quantification of Ca^2^^+^ level upon histamine stimulation. Fluorescence was monitored over time. Histamine was injected (arrow) at 50 s. Fluorescence was monitored for each individual cell over time (Fn) and normalized to the fluorescence of the first frame of the time lapse (F0). **C** The peak amplitude was the difference between the normalized fluorescence at 60 and 45 s for the Panc-1 cell line and 65 and 45 s for the MiaPaCa-2 cell line. The total number of cells for each condition was *n* = 38 (no siRNA), *n* = 25 (Myof#1 siRNA), *n* = 33 (Myof#2 siRNA) and *n* = 41 (Irrelevant siRNA) for the Panc-1 cell line and *n* = 43 (no siRNA), *n* = 79 (Myof#1 siRNA), *n* = 60 (Myof#2 siRNA) and *n* = 107 (irrelevant siRNA) for the MiaPaCa-2 cell line. Results are presented as mean ± SEM. Tukey’s test was used for statistical analysis. **** *p*-value < 0.0001. **D** Western blot validating myoferlin silencing. HSC70 was used as a loading control

Thanks to PDAC cell lines transfected with the CMV-Mito-R-GECO-1 plasmid, we monitored the modulation of mitochondrial Ca^2^^+^ levels upon histamine stimulation (Fig. 3). Since cells were in a Ca^2+^-free medium, histamine triggers Ca^2^^+^ release from internal stores, mainly through IP3Rs, allowing us to monitor Ca^2^^+^ transfer to mitochondria. To evaluate myoferlin’s impact on Ca^2^^+^ transfer, we silenced the cells for myoferlin using two siRNAs. Our results showed that upon histamine stimulation, enhanced fluorescence was visible in the no siRNA and irrelevant conditions for both Panc-1 and MiaPaCa-2 cell lines. Interestingly, in both cell lines, the cells silenced for myoferlin displayed a lower increase in fluorescence upon histamine stimulation than control conditions (no siRNA or irrelevant siRNA) (Fig. 3A).

We then monitored fluorescence over time (Fn) for each individual cell and normalized intensity to the first frame (F0) of the timelapse (Fn/F0), which allows comparisons between conditions (Fig. 3B). The quantifications confirmed the visual observations. We first notice a lower Ca^2^^+^ transfer in MiaPaCa-2 than in Panc-1 upon histamine stimulation. This difference could originate from a differential expression of *HRH1* histamine receptor. Indeed, according to protein atlas, the *HRH1* nTPM (normalized Transcripts Per Million) is 3-fold higher in PANC-1 than in MiaPaCa-2. Interestingly, mining TCGA database revealed that expression of *MYOF* gene was significantly and positively correlated to *HRH1* gene expression in cancer cell lines (Novartis/Broad dataset - rho = 0.660, *p* = 1.1 × 10^−110^, *n* = 1020) and in PDAC patients (PanCancer Database - rho = 0.661, *p* = 1.4 × 10^−23^, *n* = 184). Upon histamine stimulation, the peak of normalized fluorescence was higher in the control conditions (no siRNA and irrelevant siRNA) than in the myoferlin siRNA conditions in both cell lines. Peak amplitudes represented (Fig. 3C), were significantly lower in cells silenced for myoferlin compared with the control conditions in both Panc-1 and MiaPaCa-2 cell lines.

To our knowledge, this is the first demonstration of the role of myoferlin in histamine-triggered Ca^2^+^+^ flow into the mitochondria.

### Myoferlin silencing does not alter endoplasmic reticulum integrity

**Fig. 4:**
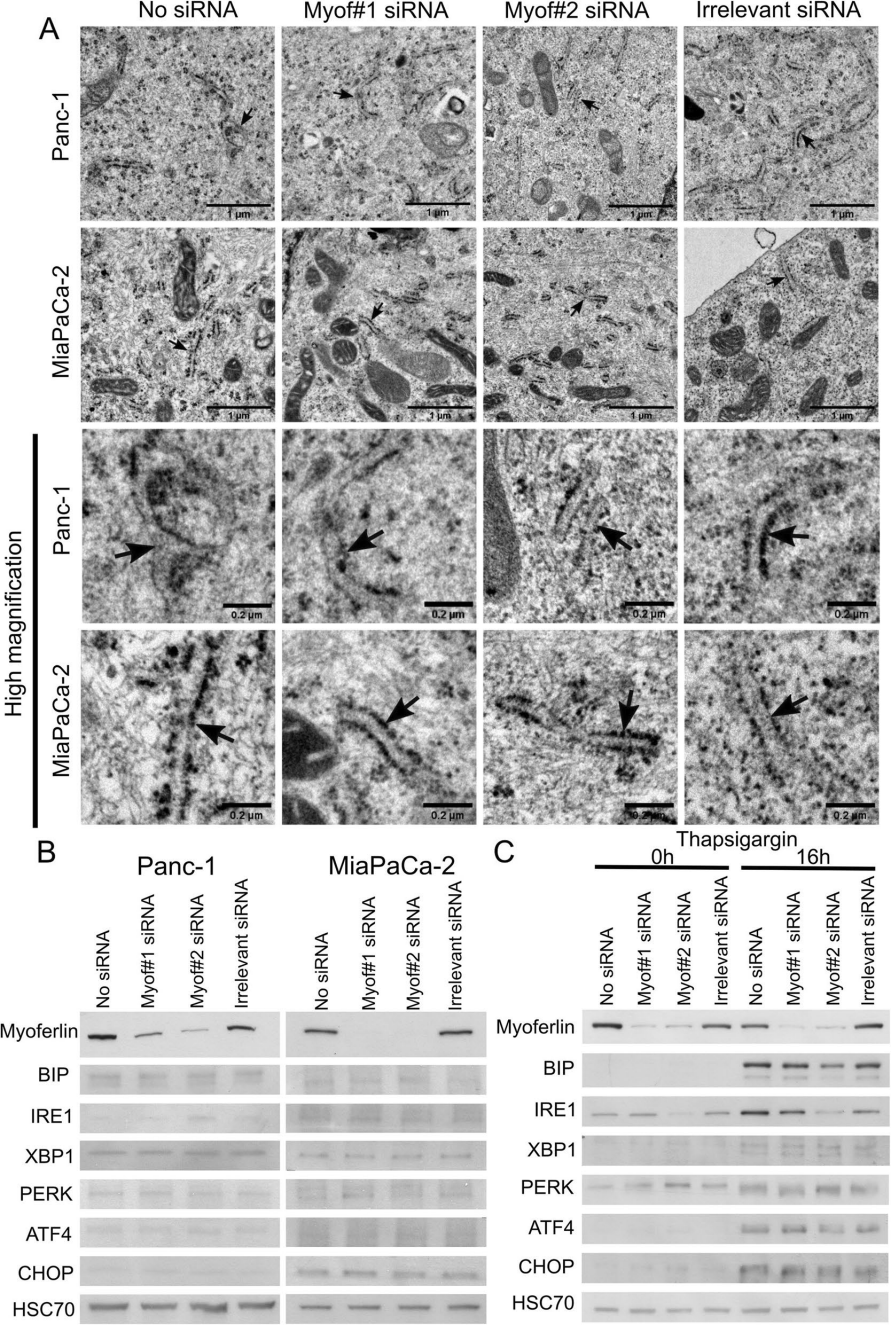
Myoferlin silencing does not alter ER morphology or induce ER stress. **A** TEM images of Panc-1 and MiaPaCa-2 cell lines. The ER is highlighted by black arrows. Scale bars = 1 μm, except for high magnification pictures where scale bars = 0.2 μm. **B** Western blot showing UPR markers upon myoferlin silencing in Panc-1 and MiaPaCa-2 cell lines. HSC70 was used as a loading control. Western blots are representative of three biological replicates. **C** Western blot showing UPR markers in Panc-1 cells silenced for myoferlin and treated with 1 µM thapsigargin

Owing to the known role of ER in Ca2+ storage, and since myoferlin has been reported to be part of the secretory pathway [33, 37], we asked whether ER integrity was altered by myoferlin silencing. Indeed, an altered ER might be not functional and unable to deliver Ca2+ to mitochondria. We first evaluated ER morphology upon myoferlin silencing based on transmission electron microscopy (TEM) images. As shown in Fig. 4A, highlighted by black arrows, no morphological alterations of the ER were visible upon myoferlin silencing in Panc-1 and MiaPaCa-2 cell lines. We saw no enlarged lumen or other ER-related abnormalities.

Additionally, to check ER Ca^2^^+^ homeostasis upon myoferlin depletion, we took advantage of the research field on ER stress. Indeed, the alteration of the ER Ca^2+^ storage triggers ER stress, inducing UPR [38]. UPR-related proteins, such as BIP, IRE1, XBP1, PERK, ATF4 or CHOP, are commonly used markers for ER stress [39]. We used these UPR markers to monitor a potential ER stress upon myoferlin silencing. None of these proteins showed increased abundance upon myoferlin depletion in Panc-1 and MiaPaCa-2 cell lines (Fig. 4B). Furthermore, myoferlin-depleted cells were still able to activate the UPR when needed. Indeed, smooth endoplasmic reticulum Ca^2^^+^ ATPase (SERCA) inhibition with thapsigargin (1 µM) significantly increased most UPR markers, even after myoferlin depletion (Fig. 4C).

According to our results, we conclude that ER structure and Ca^2^^+^ homeostasis were not impacted by myoferlin silencing.

### Myoferlin silencing does not alter abundance of MAM proteins related to Ca^2^^+^ signaling, and does not impact ER-mitochondria contact sites

**Fig. 5:**
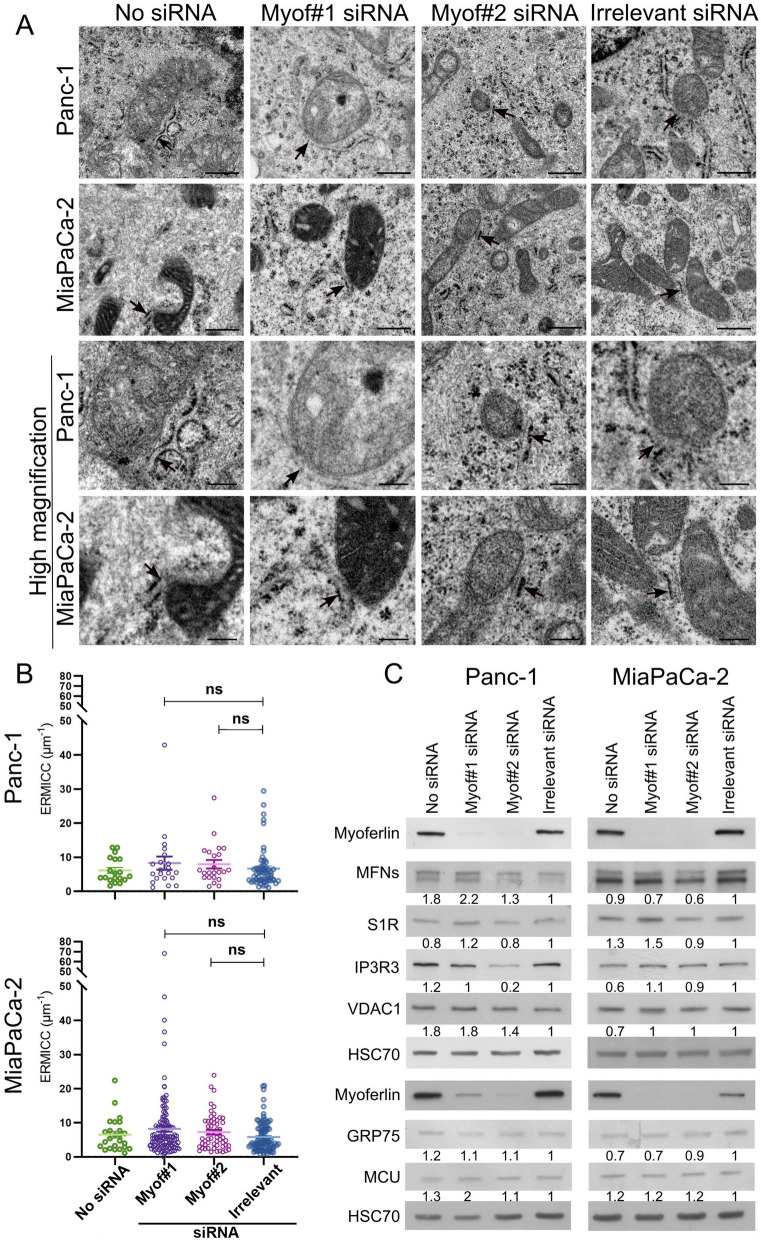
Myoferlin silencing does not impact abundance of MAMs-related proteins upon myoferlin silencing and the contacts between ER and mitochondria. **A** TEM pictures representing MAMs in Panc-1 and MiaPaCa-2 cell lines. MAMs, highlighted by black arrows on the upper panels, are shown at high magnification on the lower panel. Scale bars = 5 μm, except for high magnification, where scale bars = 0.2 μm. **B** Graphs showing the ERMICC values from controls and myoferlin-silenced cells in Panc-1 and MiaPaCa-2 cell lines. The non-parametric Kruskal-Wallis test was used for statistical analysis. For the Panc-1 cell line, the number of mitochondria in each condition was *n* = 298 (16 pictures, irrelevant), *n* = 138 (7 pictures, Myof#1 siRNA), *n* = 122 (5 pictures, Myof#5 siRNA) and *n* = 93 (6 pictures, no siRNA). Regarding the MiaPaCa-2 cell line, the number of mitochondria was *n* = 210 (10 pictures, irrelevant), *n* = 224 (9 pictures, Myof#1 siRNA), *n* = 174 (11 pictures, Myof#5 siRNA) and *n* = 189 (9 pictures, no siRNA). ns = non-significant; *: *p*-value < 0.05; **: *p*-value < 0.01. Results were presented as mean ± SEM. **C** Myoferlin, MFNs, S1R, IP3R3, VDAC1, GRP75 and MCU from whole cell lysates were assessed by western blot. The quantifications were performed with ImageJ software [24]. The irrelevant siRNA condition was used as reference for the quantifications. HSC70 was used as a loading control. The same batch of transfected Panc-1 cells was used in Fig. 6. Western blots are representative of three biological replicates

The reduced Ca^2^^+^ flow we mentioned upon myoferlin silencing could be the result of a modification of the abundance of MAMs’ proteins, mainly proteins related to Ca^2^^+^ signaling, such as IP3R3, VDAC1, MCU, S1R and GRP75. We thus evaluated the abundance of these proteins in whole cell extracts prepared from myoferlin-silenced Panc-1 and MiaPaCa-2 cell lines. Our results showed no consistent modifications upon myoferlin silencing (Fig. 5C).

Due to its ability to interact with phospholipids, we thought that myoferlin might interact with OMM phospholipids. Therefore, we hypothesized that myoferlin may modulate physical interactions between the ER and mitochondria, controlling Ca^2^^+^ transfer between organelles. Accordingly, myoferlin silencing would impair Ca^2^^+^ signaling by impacting the physical contacts between the ER and mitochondria. To validate this hypothesis, we analyzed TEM images of ER-mitochondria contact sites obtained from Panc-1 and MiaPaCa-2 cell lines (Fig. 5A) and calculate the ER-mitochondria contact coefficient (ERMICC) described by Naon et al. [28]. No significant ERMICC differences were observed in Panc-1 and MiaPaCa-2 cell lines (Fig. 5B) upon myoferlin depletion. Individual parameters are shown in the Figure S5 (Additional file 1).

**Fig. 6:**
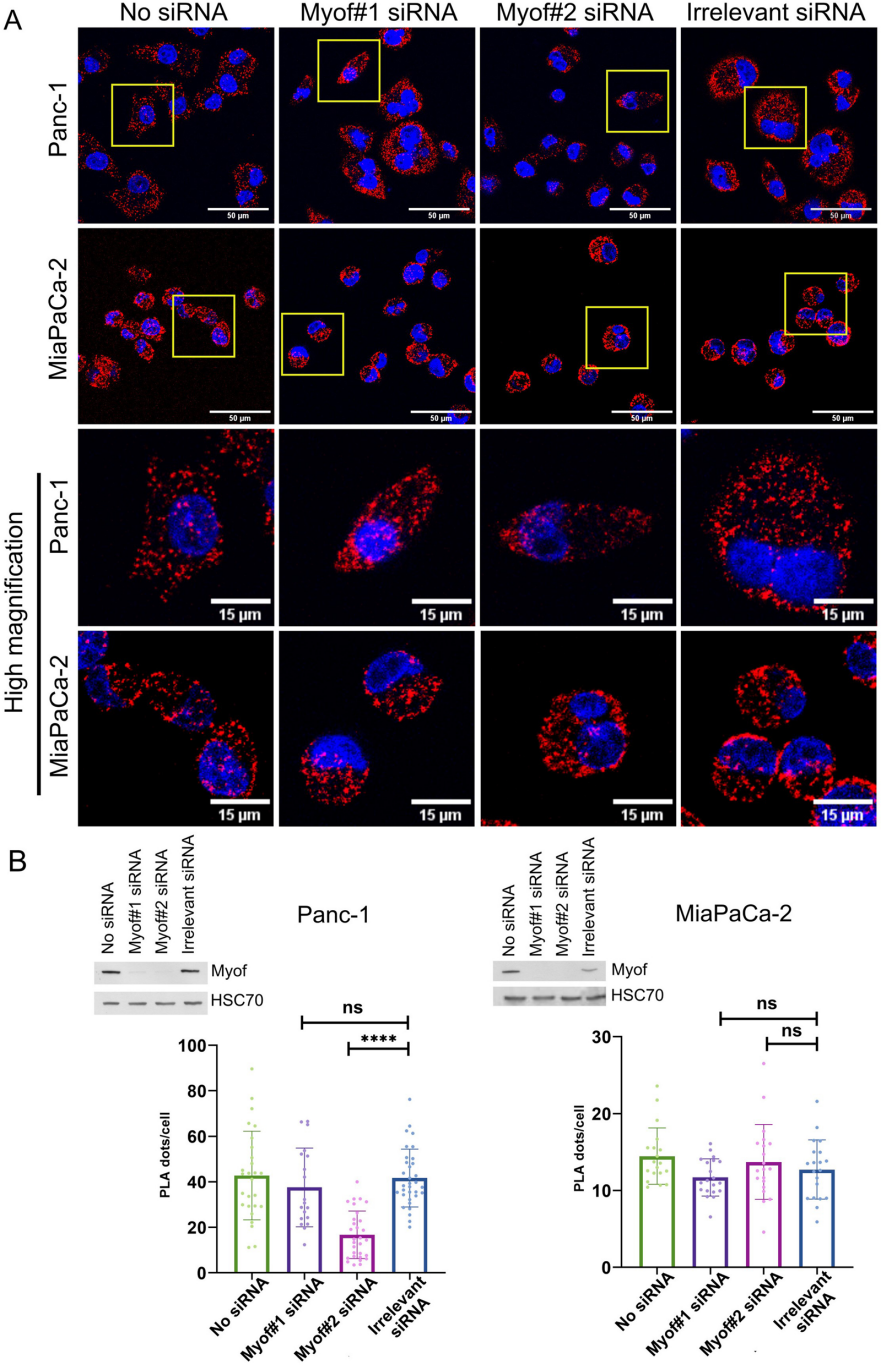
PLA between VDAC1 and IP3R3.** A** Representative pictures for PLA between VDAC1 and IP3R3 in PDAC cell lines silenced for myoferlin. The pictures were acquired with a confocal Nikon A1R microscope. **B** Quantification of PLA dots per cells in Panc-1 and MiaPaCa-2 cell lines. The number of pictures, from three independent experiments, analyzed for the PLA in the Panc-1 cell line was *n* = 33 (irrelevant), *n* = 19 (Myof#1 siRNA), *n* = 30 (Myof#2 siRNA), *n* = 27 (no siRNA). The number of pictures, from three independent experiments, for the PLA in the MiaPaCa-2 cell line was *n* = 20 (irrelevant), *n* = 20 (Myof#1 siRNA), *n* = 20 (Myof#2 siRNA) and *n* = 19 (no siRNA). Results were presented as mean ± SD. The non-parametric Kruskal-Wallis test was used for statistical analysis. **** *p*-value < 0.0001. ns: non-significant. Western blot inserts validate myoferlin silencing. HSC70 was used as a loading control. The same batch of transfected Panc-1 cells was used in Fig. 5C

In addition to the ERMICC analysis, a PLA between VDAC1 and IP3R3, two proteins forming a molecular complex that allows structural and functional coupling between ER calcium store and mitochondria [40], has been performed upon myoferlin silencing. Proximity dots were detected in both Panc-1 and MiaPaCa-2 cell lines (Fig. 6A). No significant differences were found in both cell lines regarding the number of dots per cell upon myoferlin silencing, except for the Myof#2 siRNA condition in the Panc-1 cell line, where the number of dots per cell was significantly reduced compared to the irrelevant siRNA condition (Fig. 6B). Controls for PLA are shown in Figure S6 (Additional file 1). Hereby we show that mitochondrial calcium flux disruption upon myoferlin silencing is not mediated by altered contact sites between the ER and mitochondria.

### Myoferlin interacts with IP3R3, but not with VDAC1

Since myoferlin silencing impacted neither the abundance of MAM proteins related to Ca^2^^+^ signaling, nor the ERMICC, nor the proximity between IP3R3 and VDAC1, we thought myoferlin was instead interacting with proteins involved in Ca^2^^+^ signaling in MAMs.

**Fig. 7:**
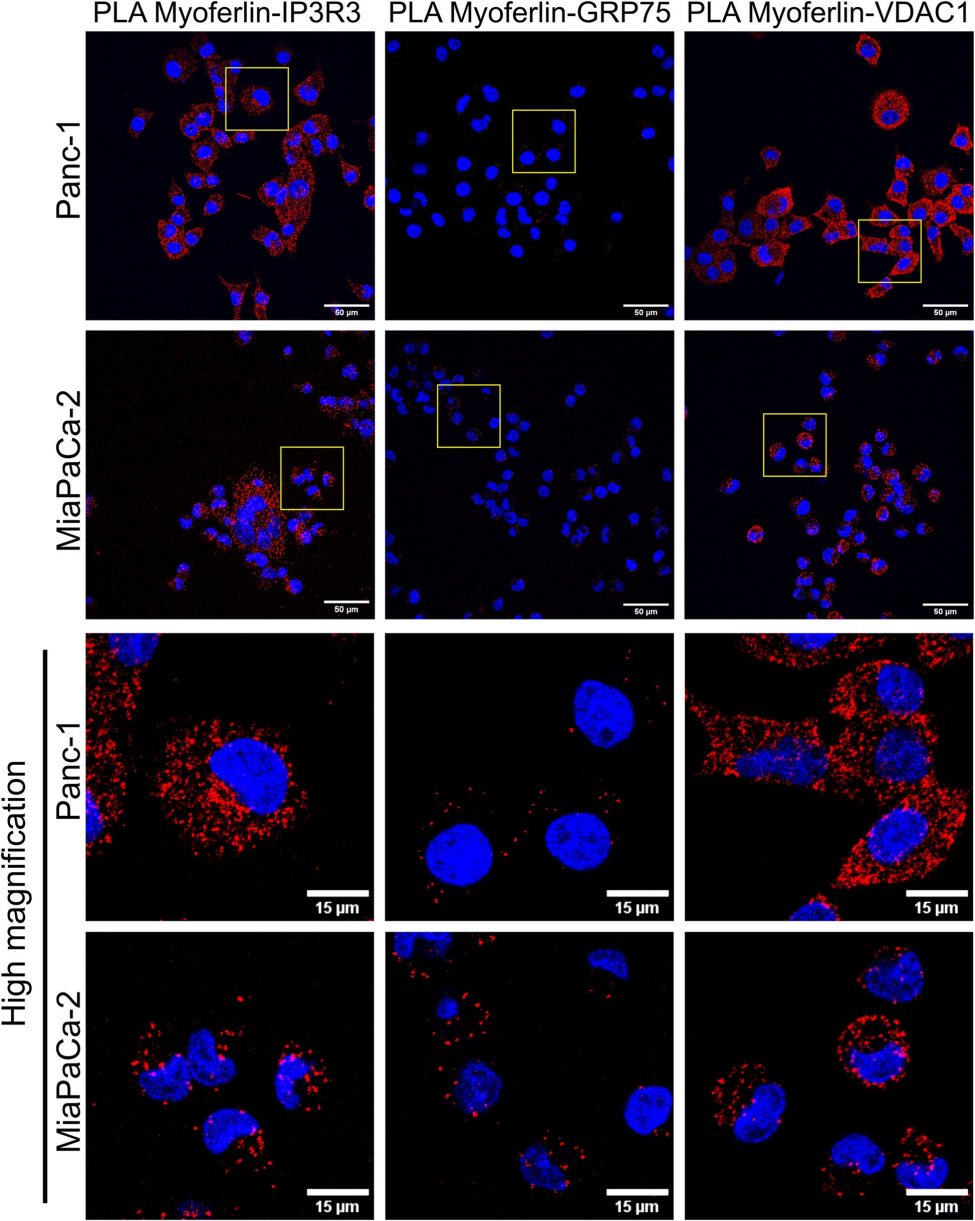
PLA between myoferlin and key proteins described in Ca^2^^+^ signaling at MAMs (IP3R3, GRP75 and VDAC1). Representative pictures for the PLA between myoferlin and IP3R3, GRP75 or VDAC1. Yellow squares are presented as high magnification in the lower panels. Pictures were acquired with Nikon A1R confocal microscope. Scale bars on the upper panel represents 50 μm, while on the lower panel it represents 15 μm. The pictures are representative of three independent experiments

To investigate our hypothesis, we performed a PLA between myoferlin and IP3R3, VDAC1 or GRP75. Interestingly, a strong proximity signal was detected between myoferlin and IP3R3 as well as between myoferlin and VDAC1 (Fig. 7). Although present, only few dots were observed for the myoferlin/GRP75 PLA. Those results demonstrated the proximity existing between myoferlin and key components of Ca^2^^+^ signaling reported in MAMs. The controls for the PLA are shown in Figure S7 (Additional file 1).

To ensure that the signals observed for the myoferlin-IP3R3 and myoferlin-VDAC1 PLA were specific, we performed the same experiment upon myoferlin silencing (Figure S8A). The number of dots per cell was drastically reduced upon silencing, indicating that the signal was specific to the proximity of myoferlin with IP3R3 or VDAC1 (Figure S8B).

**Fig. 8:**
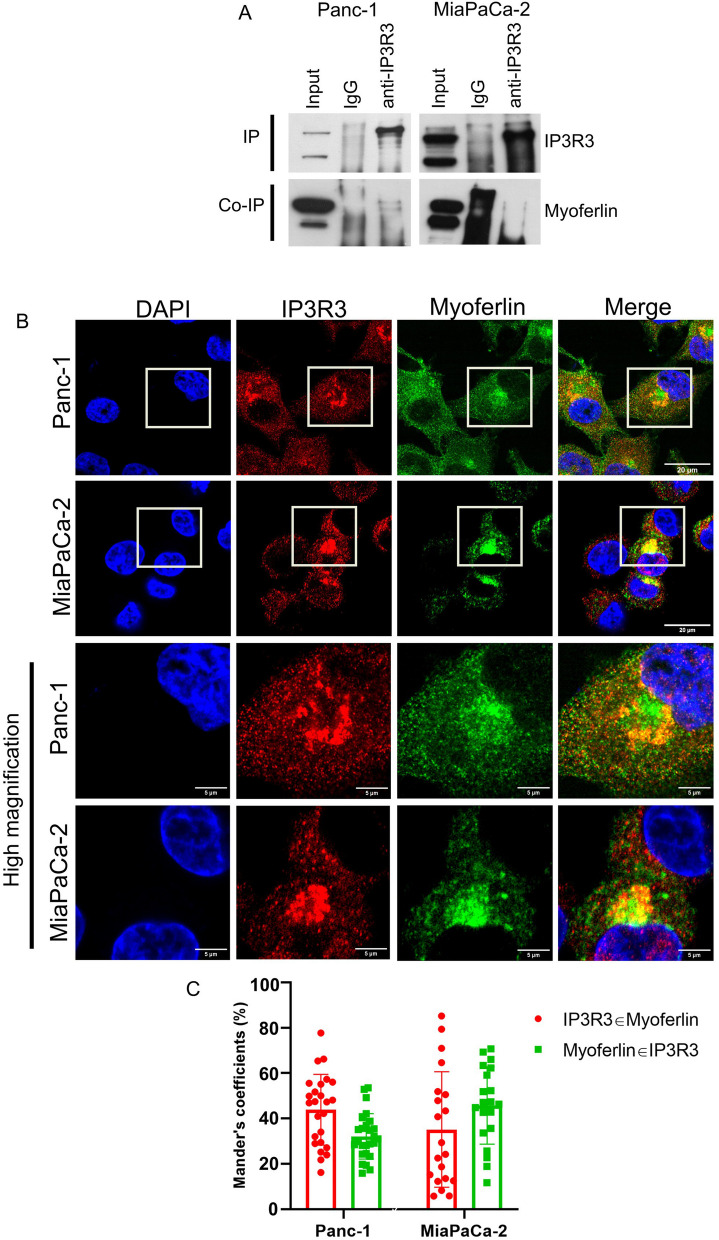
Myoferlin interacts with IP3R3. **A** IP3R3 was immunoprecipitated from Panc-1 and MiaPaCa-2 cell lines. Myoferlin co-immunoprecipitation was assessed by western blot. The IP3R3-myoferlin co-immunoprecipitation is representative of three independent experiments. **B** Myoferlin and IP3R3 immunofluorescence in Panc-1 and MiaPaCa-2 cell lines. Scale bars = 20 μm or 5 μm in high magnification. Confocal pictures were acquired with a high resolution LSM 880 microscope. **C** Colocalization analyses using Manders’ method on both Panc-1 and MiaPaCa-2 cell lines. “IP3R3 ϵ Myoferlin” represents the proportion of above-threshold pixels in IP3R3 channel colocalizing with above-threshold pixels in myoferlin channel and vice versa for “Myoferlin ϵ IP3R3”. Results are presented as mean ± SD. The number of analyzed cells was 27 for Panc-1 cells, and 20 for MiaPaCa-2. The pictures are representative of at least two independent experiments

Encouraged by these results, we decided to immunoprecipitate IP3R3 and VDAC1 from Panc-1 whole cell lysate and to check for co-immunoprecipitation of myoferlin. We efficiently immunoprecipitated IP3R3 and noticed that myoferlin was co-immunoprecipitated with this protein (Fig. 8A). Two defined bands were visible in the IP-IP3R3 condition, at the same molecular weight as the uppermost band corresponding to myoferlin in the lysate. These results prompted us to perform the same experiment on MiaPaCa-2 cell line. Similarly, we could see two bands for myoferlin as a co-immunoprecipitate of IP3R3 in this cell line (Fig. 8A). However, the bands were only barely visible in MiaPaCa-2. We could not efficiently immunoprecipitate VDAC1 and therefore, we could not conclude for myoferlin co-immunoprecipitation with this protein (results not shown).

Regarding the results obtained by co-immunoprecipitation, we were curious about a potential myoferlin-IP3R3 colocalization in the cell. To answer this question, we performed co-labeling of myoferlin and IP3R3 in both Panc-1 and MiaPaCa-2 cell lines (Fig. 8B). As previously reported, IP3R3 forms clusters at the perinuclear region [41–43] and myoferlin labeling was found throughout the cytoplasm, with a high staining intensity in the perinuclear region [33]. Interestingly, myoferlin and IP3R3 clusters seemed to colocalize in both cell lines. Then, we conducted colocalization studies. When using each individual cell as a ROI, our results showed that the proportion of IP3R3 colocalizing with myoferlin (M1) was 41.21 ± 15.38% in Panc-1 and 35.15 ± 25.46% in MiaPaCa-2 (Fig. 8C). Conversely, the proportion of myoferlin colocalizing with IP3R3 was 33.01 ± 10.88% in Panc-1 and 45.72 ± 17.04% in MiaPaCa-2 (M2).

Altogether, these results demonstrate that myoferlin is in proximity with proteins involved in Ca^2^^+^ signaling at MAMs. In addition, we were able to prove a physical interaction between myoferlin and IP3R3. Thanks to this interaction myoferlin may insure a proper Ca^2^^+^ transfer between ER and mitochondria.

### Myoferlin expression is significantly correlated with ITPR3 expression in pancreatic cancer but not in normal pancreas

To further investigate the significance of our discoveries, we exploited the ARCHS4 mining tool [44] to explore the potential correlation between myoferlin and *IP3R3* gene expressions. Thanks to the correlation AnalyserR tool [45], we assessed Pearson’s correlation between *MYOF* and every gene of the genome in healthy (Additional file 2) and tumoral (Additional file 3) conditions.

**Fig. 9:**
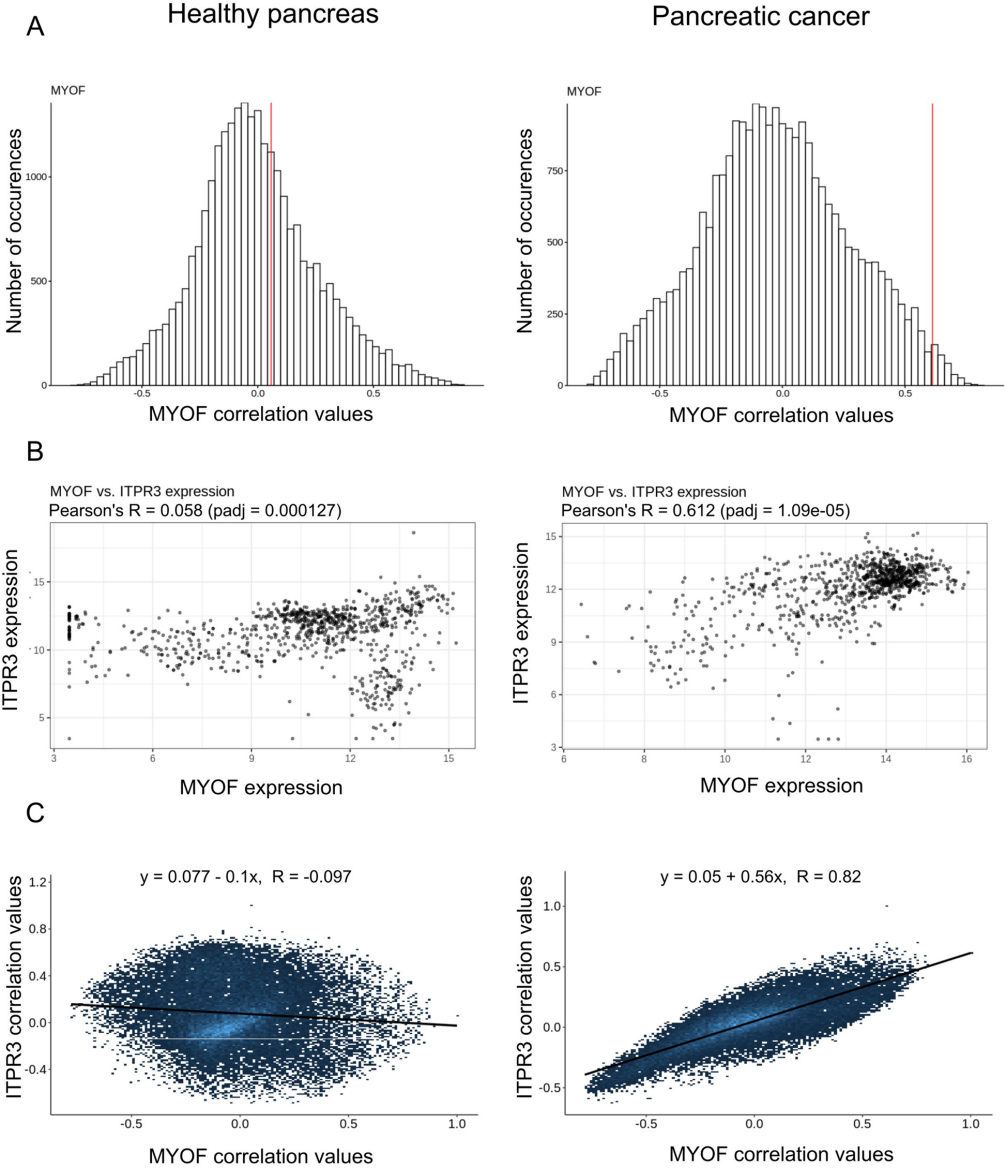
Correlation of *MYOF* expression with *ITPR3* expression in normal and cancer pancreas.** A** Distribution of correlation coefficient between every gene of the genome and *MYOF* in normal and cancer pancreas. The red vertical lines represented are the correlation values between *ITPR3* and *MYOF*. **B** Scatterplots of the expressions of *MYOF* and *ITPR3* genes are represented. **C** Correlation of gene expression with *ITPR3* expression according to its correlation with *MYOF* gene expression

The distribution of the binned Pearson coefficient was established (Fig. 9A). Each bin represents the number of genes that were found to be correlated with *MYOF* with a coefficient within this bin. In healthy condition, *MYOF* and *ITPR3* expressions were not correlated (*R* = 0.058, *p*-value: 0.0017), while in pancreatic cancer their expression appeared strongly and significantly correlated (*R* = 0.612, *p*-value: 1.09e-5) (Fig. 9B). The GEO identification numbers of the samples used for this analysis are listed in Additional file 4. There are 904 samples for normal pancreas and 961 samples for cancer pancreas, all are included in the GEO database. We then depicted the correlation between the expression of individual genes with *ITPR3* expression as a function of its correlation with *MYOF* expression (Fig. 9C). In this plot, each point corresponds to an individual gene. The 23,478 genes used for the analysis along with their correlation with *MYOF* and *ITPR3* are listed in Additional files 5 and 6 for normal and cancer pancreas, respectively. We noticed that genes correlated to *ITPR3* expression in pancreatic cancer are also correlated to *MYOF* expression (*R* = 0.82), while it was not the case in healthy pancreas (*R*=-0.097). In cancer, the same genes are thus simultaneously correlated with *MYOF* and *ITPR3*, tending to show that *MYOF* and *ITPR3* are involved in the same cellular pathways.

## Discussion

Myoferlin has been shown to influence mitochondrial respiration and network in pancreatic and breast cancers [46, 47]. Nevertheless, the mechanism by which myoferlin silencing impacts mitochondrial metabolism and dynamics is still unknown. As a first attempt to understand the mechanisms by which myoferlin influences mitochondria, we aimed at clarifying myoferlin localization in relation to this organelle. In this report, we demonstrated that myoferlin is not located on mitochondria of PDAC cell lines, but rather in MAMs. Our findings suggest that myoferlin may influence mitochondrial function thanks to this specific location.

Myoferlin localization in MAMs is supported by previous reports. Indeed, several proteomic studies have been performed on MAMs from various tissues and species [48–53]. In human, myoferlin was detected in MAMs isolated from skin fibroblasts, skeletal muscles, testis and liver [48, 49, 51, 53], while myoferlin paralog, dysferlin, is found to MAMs from liver and skeletal muscles [49, 52, 53]. In accordance with our findings, organelle-specific proteomic analyses did not detect myoferlin in human mitochondria [54–56]. Further investigations looking at the ferlin family members in MAMs could be of interest, opening new insights about ferlin functions.

In addition to the discovery of myoferlin as a MAMs component, we found that silencing this protein impairs the histamine-triggered Ca^2^^+^ transfer to mitochondria. Owing to the reported increase of PDAC cell migration upon histamine treatment, and its correlation with Ca^2^^+^ mobilisation [57], it can be hypothesized that the previously reported alteration of PDAC cell migration by myoferlin silencing [9] occurs through the decrease of Ca^2^^+^ transfer. To explain the involvement of myoferlin in the Ca^2^^+^ transfer to mitochondria, we highlighted a proximity existing between myoferlin and key proteins involved in Ca^2^^+^ signaling at MAMs, such as IP3R3, VDAC1 and, to a lower extent, GRP75. Based on our results and on the literature, we suggest that myoferlin could be involved in a protein complex located in MAMs. Indeed, Szczesniak et al. [58] identified myoferlin as being part of the interactome of the BCL-2 related ovarian killer (BOK), a protein involved in Ca^2^^+^ signaling at MAMs. This member of the BCL-2 family interacts and protects IP3Rs from degradation [58]. Moreover, myoferlin has been shown to interact with STAT3, which acts as a gatekeeper for ER-mitochondria Ca^2^^+^ fluxes [59–61]. Indeed, Su et al. [62] showed that STAT3 was probably located in MAMs rather than on mitochondria, in opposition with the commonly accepted paradigm. In our study, we observed strong signals for the PLA between myoferlin and VDAC1. While we failed at co-immunoprecipitating VDAC1 and myoferlin, myoferlin interaction with VDAC1 has been previously reported [63]. In addition, myoferlin has been reported as being part of a protein complex involving RAB32, a protein described as a MAMs component, playing a role in mitochondrial fission [13]. RAB32 prevents mitochondrial fission by phosphorylation of DRP1 and its silencing results in mitochondrial fragmentation and a decreased mitochondrial respiration. Myoferlin pharmacological targeting has been shown to dissociate the RAB32-myoferlin complexes, which has been proposed as a mechanism explaining the impact of myoferlin pharmacological targeting on mitochondria [13]. However, even if myoferlin has been described as being part of a protein complex with RAB32, the relationship/localization between RAB32 and myoferlin remained unclear. Our work might suggest that this interaction occur at the interface of mitochondria with other organelles.

Myoferlin has been reported on various localizations in the cell such as plasma membrane, late and early endosomes, lysosomes, exosomes, Golgi apparatus and ER [33, 64–66]. Therefore, the proximity observed between myoferlin and VDAC1, IP3R3 or GRP75 could reflect several localizations of myoferlin within the cell. For instance, myoferlin has been described on lysosomes of PDAC cell lines [65]. Since lysosomes have been shown to be in contact with mitochondria and ER, it is conceivable that those contacts participate also to the observations presented in this work [67–70]. Indeed, it is known that Ca^2^^+^ transfer also occurs between lysosomes and ER as well as mitochondria [68, 69].

## Conclusions

In this work, we aimed at clarifying myoferlin localization in relation to mitochondria. For the first time, we demonstrated that myoferlin is not located on mitochondria of PDAC cell lines, but rather in MAMs where it insures proper Ca^2^^+^ transfer to mitochondria. This discovery is of special interest since the recent discovery of the involvement of mitochondrial Ca2+ in migration, invasion, metastasis, and metabolic stress resistance of PDAC cells [71].

### Supplementary Information

**Additional file 1.**

** Additional file 2.**

** Additional file 3.**

** Additional file 4.**

** Additional file 5.**

** Additional file 6.**

## Data Availability

No datasets were generated or analysed during the current study.

## Abbreviations

ATF4: Activating transcription factor 4
BIP: Binding immunoglobulin protein
BOK: BCL-2 related ovarian killer
CHOP: Transcription factor C/EBP- homologous protein
CM: Crude mitochondrial fraction
CMAMs: Crude MAMs
COXIV: Cytochrome c oxidase subunit 4
DistER-M: Distance between mitochondria and ER
DMEM: Dulbecco’s modified Eagle’s medium
ER: Endoplasmic reticulum
ERMICC: ER-mitochondria contact coefficient
FBS: Fetal bovine serum
GAPDH: Glyceraldehyde-3-phosphate dehydrogenase
GLUT1: Glucose transporter 1
GRP75: 75 KDa glucose- regulated protein
GRP78: 78-kDa glucose-regulated protein
HSC70: Heat shock cognate 71 kDa protein
IMM: Inner mitochondrial membrane
IP3R3: Inositol 1,4,5-triphosphate receptors 3
IRE1: Inositol requiring enzyme 1
Lin: length of ER-mitochondria interface
MAMs: Mitochondria-associated membranes
MERCS: Mitochondria-contact sites
MFN1/2: Mitofusin 1/2
OMM: Outer mitochondrial membrane
PDAC: Pancreatic ductal adenocarcinoma
PERK: PKR-like ER kinase
PerM: Mitochondrial perimeter
PLA: Proximity ligation assay
PM: Purified mitochondrial fraction
PMAMs: Purified MAMs
ROI: Region of interest
RT: Room temperature
S1R: Sigma-1 receptor
SDS: Sodium dodecyl sulfate
siRNA: Small interfering RNA
SP1: Specificity protein 1
TEM: Transmission electron microscopy
TOM20: Mitochondrial import receptor subunit TOM20 homolog
UPR: Unfolded protein response
VDAC1: Voltage-dependent anion channel 1
XBP1s: X-box-binding protein-1
