# Supplementary material for: Identification of myoferlin as a mitochondria-associated membranes component required for calcium signaling in PDAC cell lines
Source: Cell Commun Signal. 2024 Feb 17;22:133. doi: 10.1186/s12964-024-01514-z (PMC10874564; doi:10.1186/s12964-024-01514-z)
Supplement: Supplementary file 1 — Additional file 1. [file 12964_2024_1514_MOESM1_ESM.pdf]

Figure S1

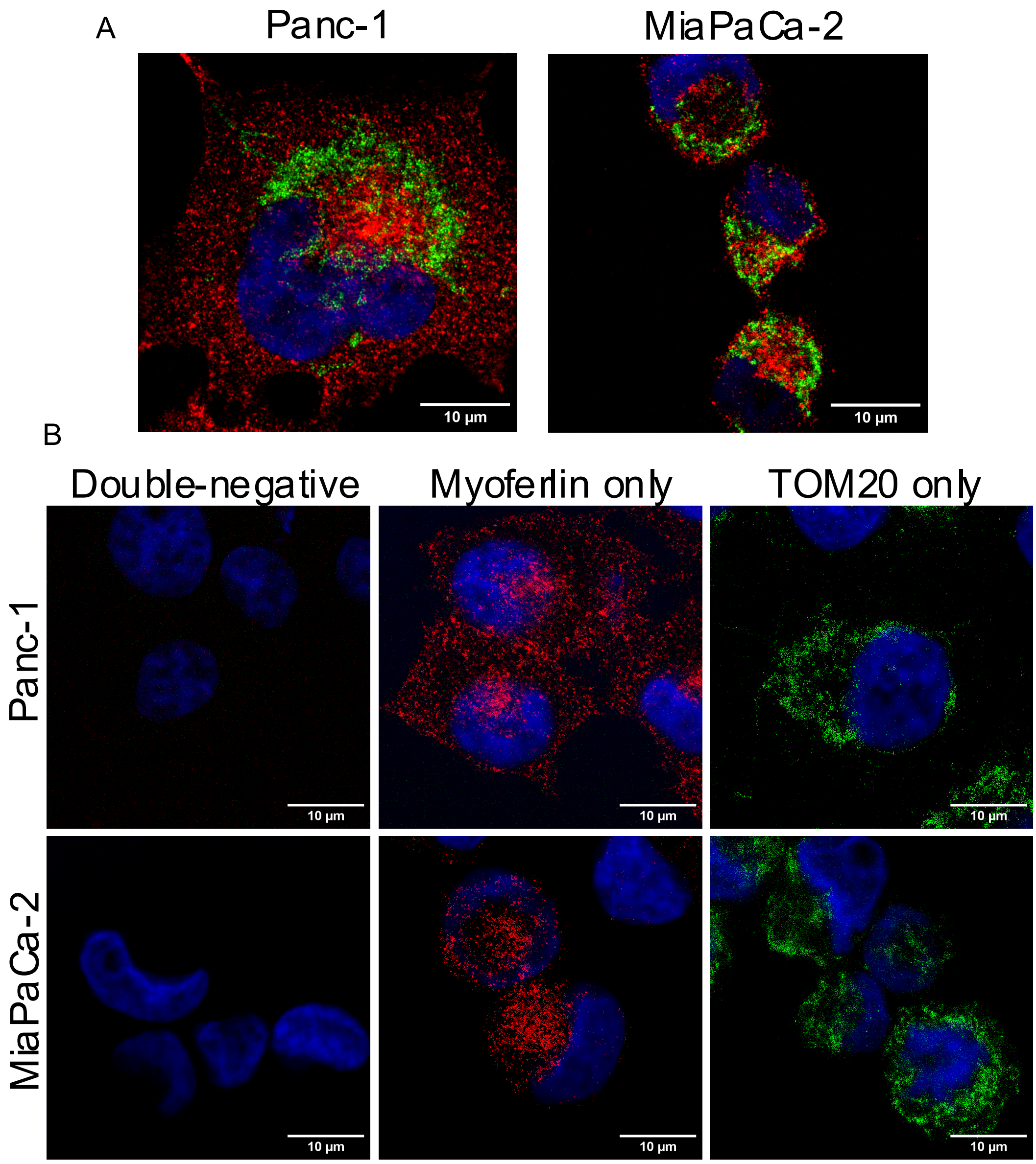

Figure S1. **(A)** Pictures at low magnification of myoferlin-TOM20 co-staining in Panc-1 and MiaPaCa-2 cell lines. **(B)** Controls for the myoferlin-TOM20 co-staining. No primary antibodies (double-negative control), myoferlin-only primary antibody or TOM20-only primary antibody were used as negative controls in the presence of secondary antibodies. The negative controls were performed on both Panc-1 and MiaPaCa-2 cell lines.

Figure S2

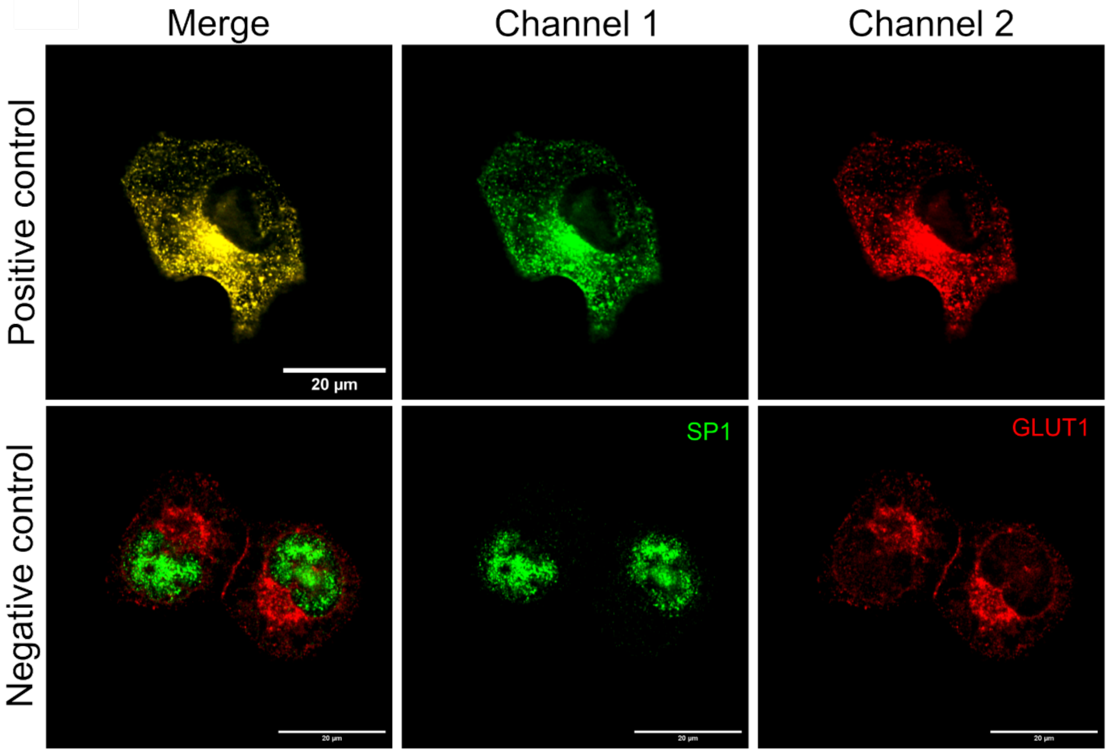

Figure S2. Example of pictures for positive and negative controls. Positive control was performed on Panc-1 cells. Two secondary antibodies carrying distinct fluorochromes (Alexa Fluor 488 and 546), recognized the same myoferlin rabbit polyclonal primary antibody (HPA). Negative control with SP1 in green and GLUT1 in red. Confocal pictures were acquired with a high-resolution LSM 880 microscope.

Figure S3

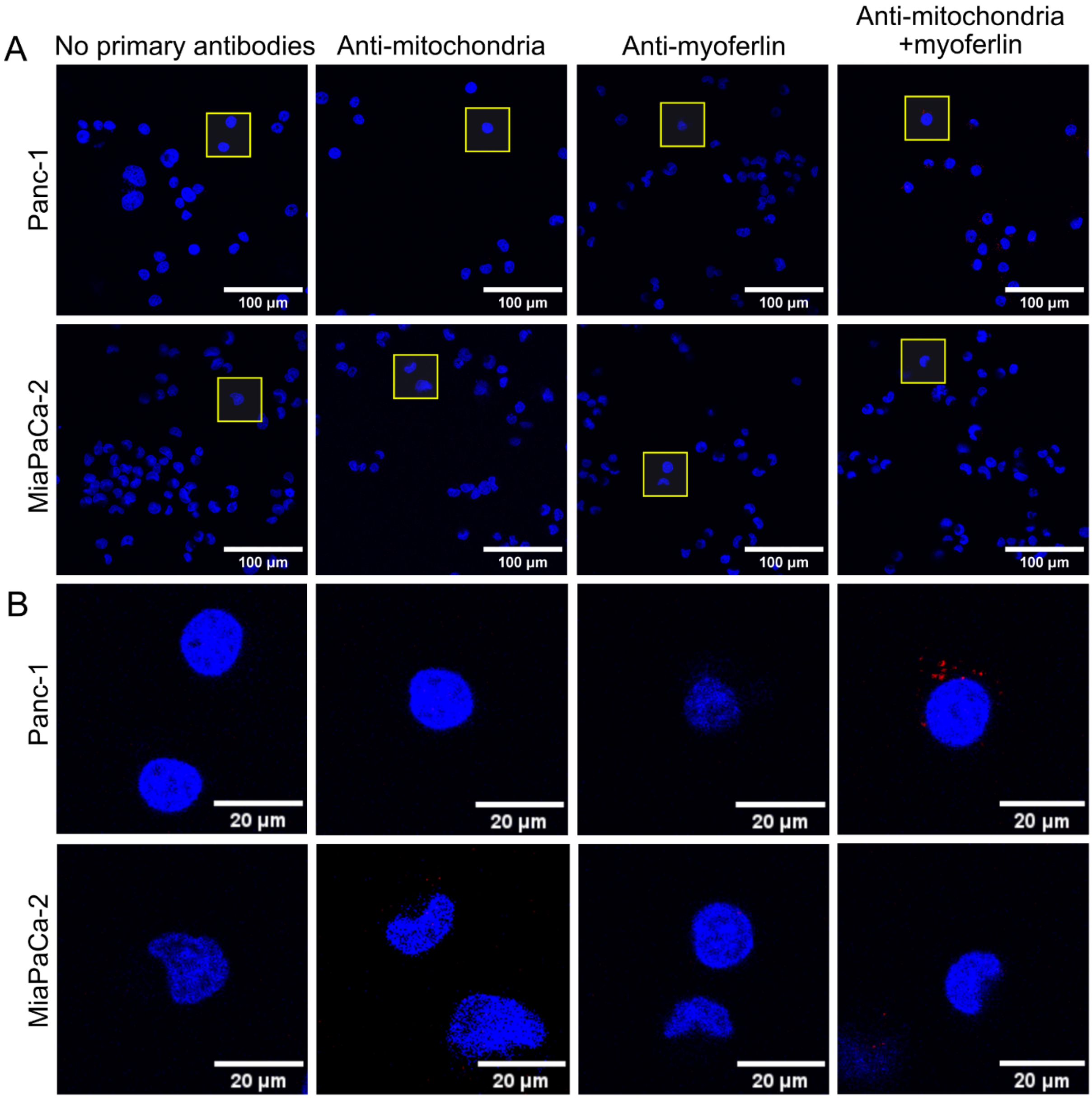

Figure S3. Myoferlin is not close to a 60 KDa OMM protein and is only barely present in purified mitochondria. **(A)** Representative pictures of PLA obtained from samples incubated without primary antibodies (no primary antibodies), with only one primary antibody (60 kDa OMM protein or myoferlin), or with both primary antibodies. Yellow squares are represented in **(B)** at higher magnification. Pictures were acquired using a confocal Nikon A1R microscope. The experiment was performed twice.

Figure S4

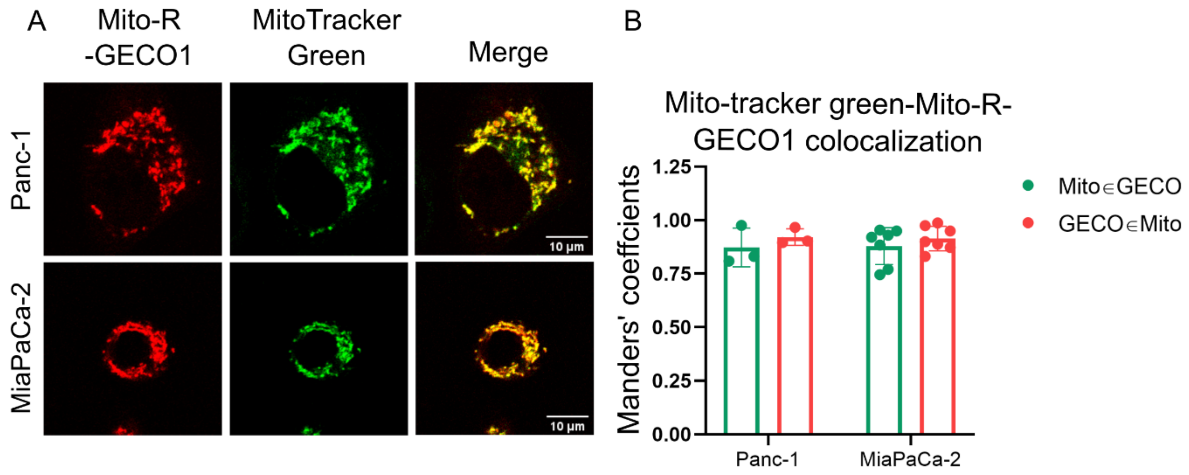

Figure S4. Validation of mitochondrial localization for the fusion protein encoded by the CMV-Mito-R-GECO-1 plasmid in Panc-1 and MiaPaCa-2 cell lines. **(A)** The fusion protein appears in red (RFP), while the mitochondrial probe (MitoTracker) is in green. The fusion protein and the MitoTracker green probe are perfectly colocalizing (Merge picture, yellow pixels). **(B)** Colocalization analysis using Manders' method. One dot represents one cell. The Mito ϵ GECO (in green on the graph) represents the proportion of above-threshold pixels for the MitoTracker green probe colocalizing with above-threshold pixels for the CMV-Mito-R-GECO-1 fusion protein. The opposite is represented by the "GECO ϵ Mito" (in red on the graph).

Figure S5

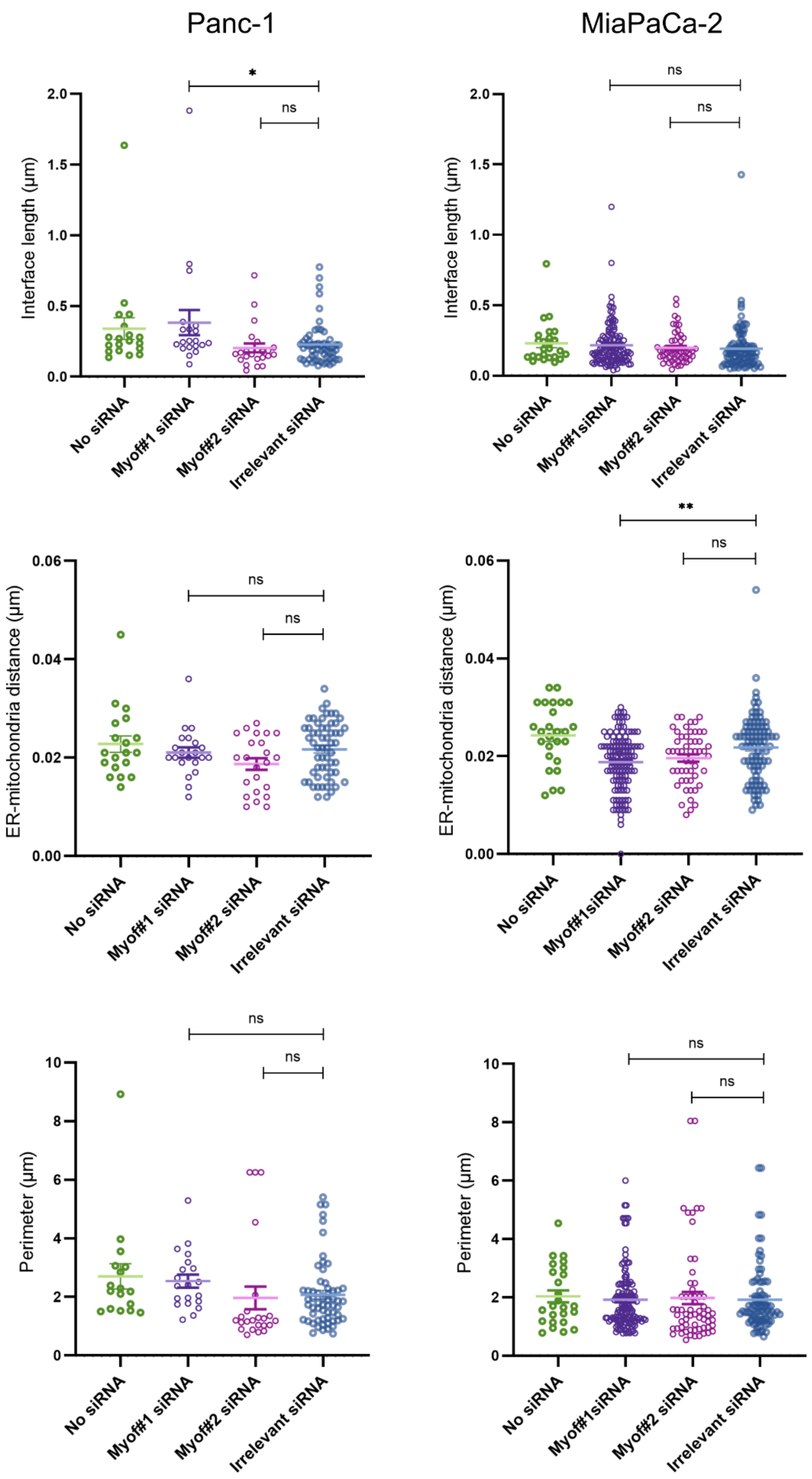
Panc-1
MiaPaCa-2
Interface length (µm)
ER-mitochondria distance (µm)
Perimeter (µm)
No siRNA
Myof#1 siRNA
Myof#2 siRNA
Irrelevant siRNA
Myof#1siRNA
*
**
ns

Figure S5. Myoferlin silencing does not impact the topology of contacts between ER and mitochondria in Panc-1 and MiaPaCa-2 cell lines. **(A)** Interface length of ER in contact with mitochondria in controls and myoferlin-silenced cells. **(B)** Distance between ER and mitochondria in controls and myoferlin-silenced cells. **(C)** Graphs showing the perimeter of mitochondria in contact with ER in controls and myoferlin-silenced cells. The non-parametric test of Kruskal-Wallis was used for statistical analysis. For the Panc-1 cell line, the number of mitochondria in each condition was: n=298 (16 pictures, irrelevant), n=138 (7 pictures, Myof#1 siRNA), n=122 (5 pictures, Myof#2 siRNA), and n=93 (6 pictures, no siRNA). Regarding the MiaPaCa-2 cell line, the number of mitochondria was: n=210 (10 pictures, irrelevant), n= 224 (9 pictures, Myof#1 siRNA), n=174 (11 pictures, Myof#2 siRNA), and n= 189 (9 pictures, no siRNA). ns = non-significant. *: p-value < 0.05. **: p-value < 0.01. Mean ±SEM is represented.

Figure S6

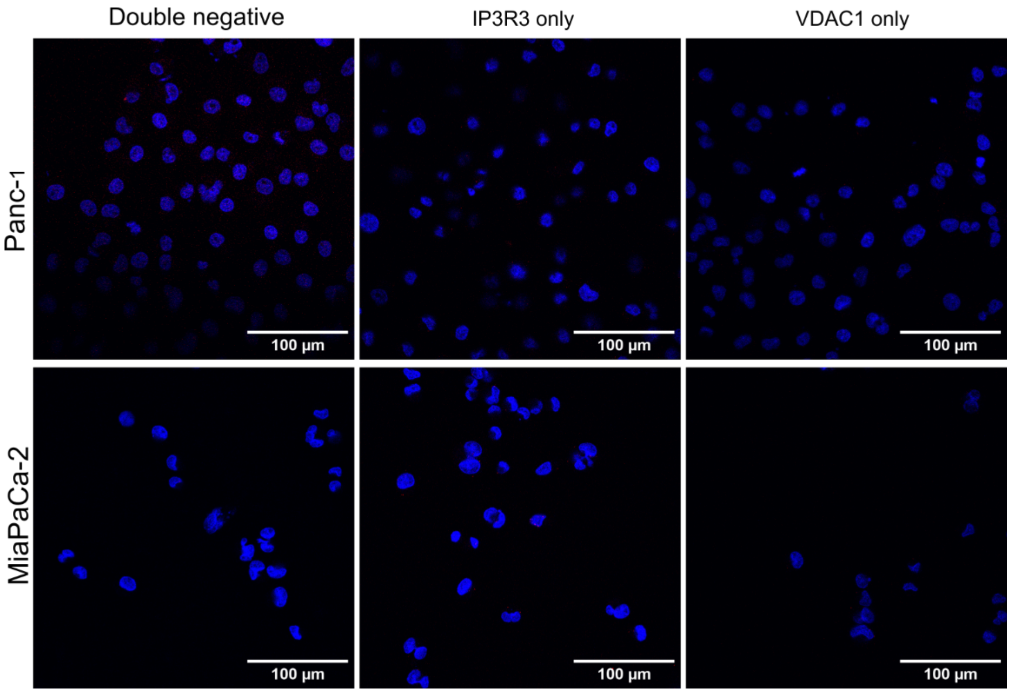

Figure S6. Negative controls for the PLA between IP3R3 and VDAC1 in Panc-1 and MiaPaCa-2 cell lines. No primary antibodies (double-negative control), IP3R3-only primary antibody or VDAC1-only primary antibody were used as negative controls in the presence of secondary antibodies.

Figure S7

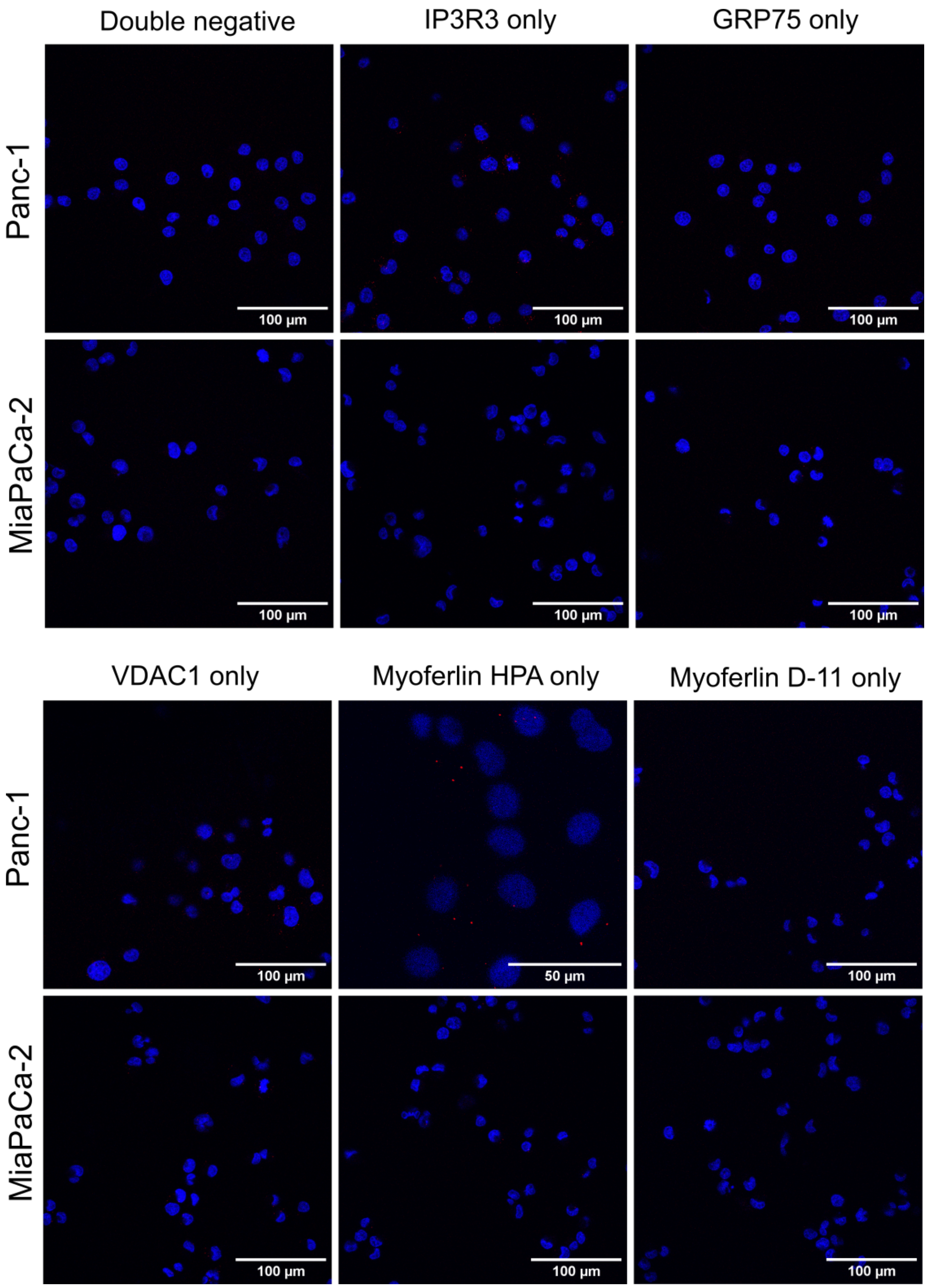

Figure S7. Negative controls for the PLA between myoferlin and IP3R3, GRP75 or VDAC1 in Panc-1 and MiaPaCa-2 cell lines. No primary antibodies (double-negative control), IP3R3-only primary antibody, GRP75-only primary antibody, VDAC1-only primary antibody or myoferlin (D-11 or HPA, depending on species compatibility)-only primary antibody were used as negative controls in the presence of secondary antibodies.

Figure S8

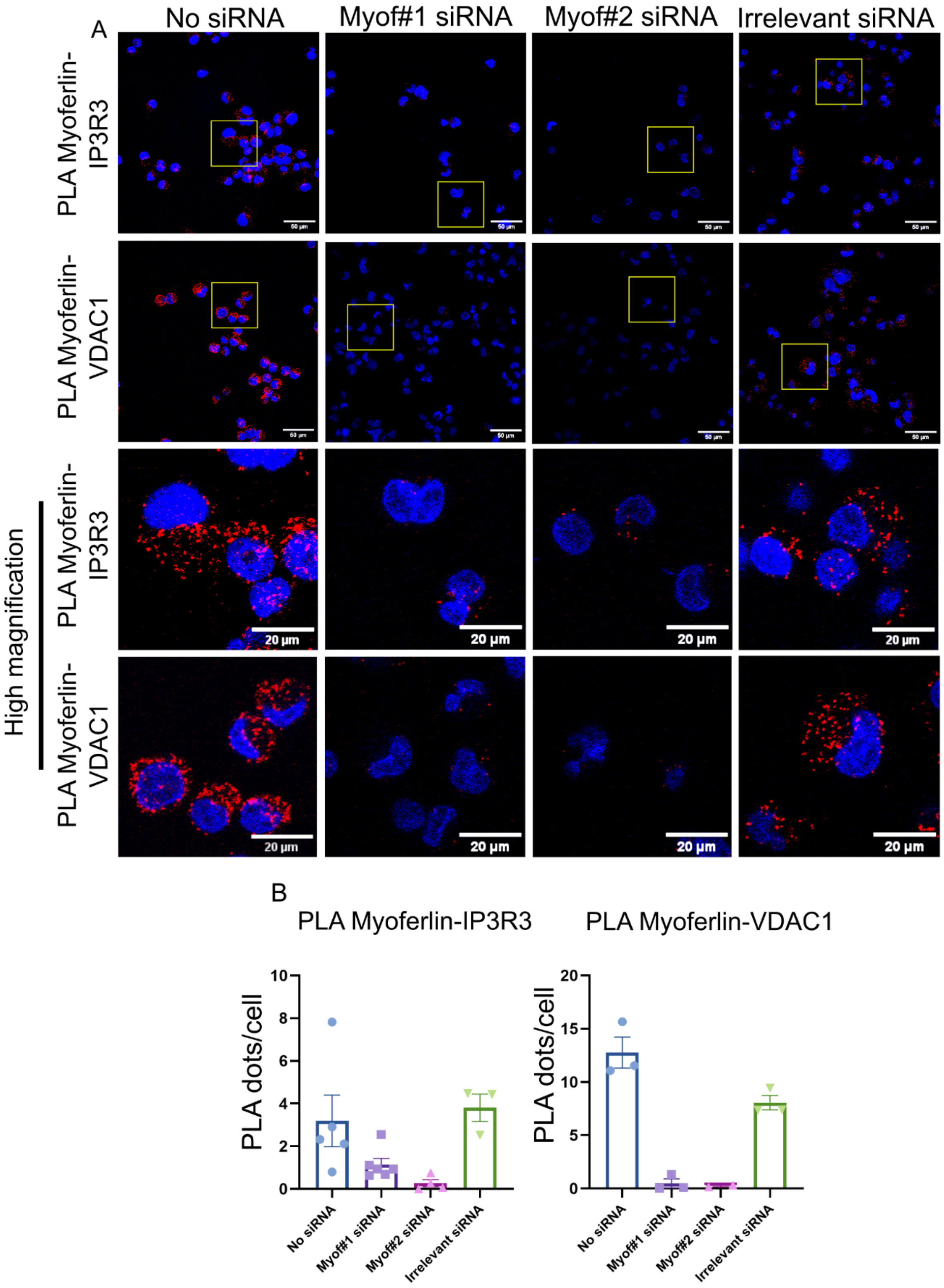

Figure S8. (A) Representative pictures of PLA for myoferlin-VDAC1 or myoferlin-IP3R3 upon myoferlin silencing. Scale bars = 50 µm (upper panel), scale bars = 20 µm (lower panel). Pictures were acquired with a Nikon A1R microscope. **(B)** Quantification of PLA dots per cell for both myoferlin-IP3R3 and myoferlin-VDAC1 PLA.

Table S1

Table S1: Descriptive statistics related to Figure 1C. Cell type, Average (Mean) percentage of colocalization, standard deviation (SD), sample size (n), and p-values.

	Cell type	*Mean*	*SD*	*n*	*p-value*
Colocalization Myof-TOM20	Panc-1	12.82 (M1)<br>7.160 (M2)	5.016 (M1)<br>3,560 (M2)	55	0.7674 (M1)<br>0.1133 (M2)
	MiaPaCa-2	7.708 (M1)<br>7.661 (M2)	5,612 (M1)<br>6,321 (M2)	114	0.9999 (M1)<br>0.0845 (M2)
Negative control	Panc-1	12.81 (M1)<br>3.929 (M2)	12,00 (M1)<br>2,960 (M2)	24	
Positive control	Panc-1	87.17 (M1)<br>99.83 (M2)	2,716 (M1)<br>0,094 (M2)	16	
